# Potential Anti-SARS-CoV-2 Therapeutics That Target the Post-Entry Stages of the Viral Life Cycle: A Comprehensive Review

**DOI:** 10.3390/v12101092

**Published:** 2020-09-26

**Authors:** Rami A. Al-Horani, Srabani Kar

**Affiliations:** Division of Basic Pharmaceutical Sciences, College of Pharmacy, Xavier University of Louisiana, New Orleans, LA 70125, USA; skar@xula.edu

**Keywords:** COVID-19, SARS-CoV-2, main protease, papain-like protease, RNA-dependent RNA polymerase, dihydroorotate dehydrogenase, remdesivir, dexamethasone, favipiravir, EIDD-2801

## Abstract

The coronavirus disease-2019 (COVID-19) pandemic continues to challenge health care systems around the world. Scientists and pharmaceutical companies have promptly responded by advancing potential therapeutics into clinical trials at an exponential rate. Initial encouraging results have been realized using remdesivir and dexamethasone. Yet, the research continues so as to identify better clinically relevant therapeutics that act either as prophylactics to prevent the infection or as treatments to limit the severity of COVID-19 and substantially decrease the mortality rate. Previously, we reviewed the potential therapeutics in clinical trials that block the early stage of the viral life cycle. In this review, we summarize potential anti-COVID-19 therapeutics that block/inhibit the post-entry stages of the viral life cycle. The review presents not only the chemical structures and mechanisms of the potential therapeutics under clinical investigation, i.e., listed in clinicaltrials.gov, but it also describes the relevant results of clinical trials. Their anti-inflammatory/immune-modulatory effects are also described. The reviewed therapeutics include small molecules, polypeptides, and monoclonal antibodies. At the molecular level, the therapeutics target viral proteins or processes that facilitate the post-entry stages of the viral infection. Frequent targets are the viral RNA-dependent RNA polymerase (RdRp) and the viral proteases such as papain-like protease (PL^pro^) and main protease (M^pro^). Overall, we aim at presenting up-to-date details of anti-COVID-19 therapeutics so as to catalyze their potential effective use in fighting the pandemic.

## 1. Introduction

Over the last 20 years, humanity has dealt with three serious coronavirus infection outbreaks, namely severe acute respiratory syndrome coronavirus (SARS-CoV, 2002–2003) [1], Middle East respiratory syndrome coronavirus (MERS-CoV, 2012–2019) [2], and SARS-CoV-2, (2019-present) [3]. Although it appears that the fatality rates for the first two outbreaks are much higher (9.2% and 37%, respectively) than the ongoing pandemic (~3.3% as of 5 September 2020) [4,5], the ongoing infectious disease of SARS-CoV-2 appears to be far more contagious. The ongoing outbreak, widely known as coronavirus disease of 2019 (COVID-19), was recognized by the World Health Organization as a global pandemic on 11 March 2020 [6]. As of 5 September 2020, there have been more than 26.7 million confirmed cases worldwide with more than 876 thousand global deaths [4].

Efforts are ongoing to deliver an effective vaccine to protect individuals against the disease. Likewise, potential therapeutics to prevent and/or treat the disease and its complications are being advanced to clinical trials all around the world. In this direction, effective treatments for COVID-19 patients, particularly those who have the severe version of the disease and become critically ill needing hospitalization, intensive care unit (ICU) admission, and mechanical ventilation, appear to include antiviral drugs as well as anti-inflammatory drugs and anticoagulant drugs to also treat the associated cytokine storm [7] and coagulopathies [8], respectively.

Considering the current clinical guidelines, remdesivir has been recommended for the treatment of COVID-19 in hospitalized patients with severe disease [9]. Furthermore, favipiravir has been approved for the treatment of COVID-19 in the hospital settings in few countries [10]. Moreover, dexamethasone as an anti-inflammatory drug has also been recommended in patients with COVID-19 who require mechanical ventilation or supplemental oxygen [11]. Despite the above recommendations and/or approvals, the need for effective treatment remains largely unmet. Therefore, a large number of potential therapeutics continue to be developed and others are being advanced into clinical trials. We recently reviewed the chemical and mechanistic aspects of antiviral drugs that block the early phase of the virus life cycle [12]. In this article, we review the chemical structures and the mechanisms of action of potential antiviral therapeutics that block/inhibit the post-entry stages of the virus life cycle. We only include those therapeutics that are listed in clinicatrials.gov. They include both old drugs and new molecular entities. Many of the potential therapeutics are small molecules and few are macromolecules. Some of these therapeutics also possess anti-inflammatory effects.

### The Life Cycle of SARS-COV-2 and Potential Targets for Drug Development

**Figure 1:**
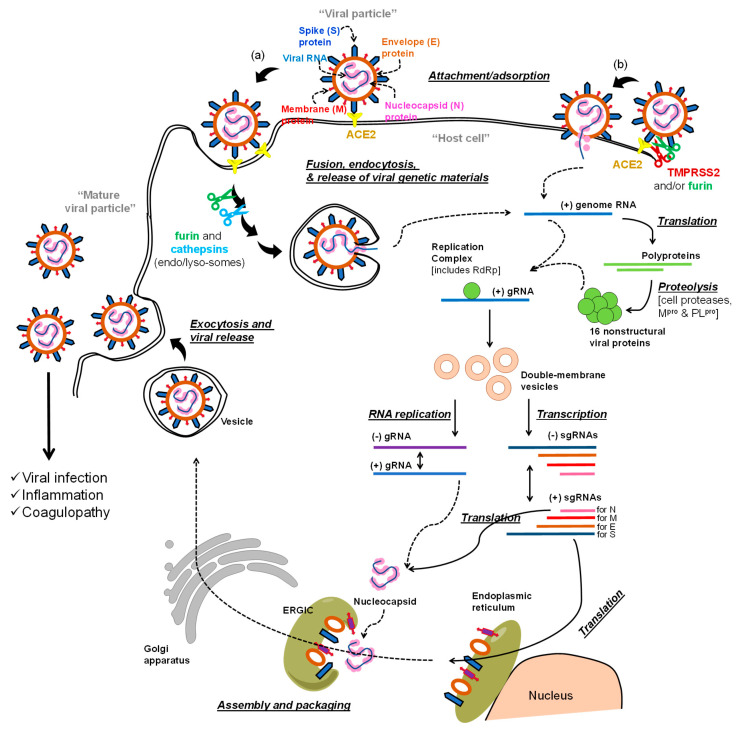
Representation of the viral particle of SARS-CoV-2 demonstrating the structural proteins: S (blue), E (brownish orange), M (red), and N (pink). It also details the post-entry stages of the life cycle of the virus. Following the release of the viral RNA genome to the host cell environment, the nonstructural proteins (NSPs) domain is expressed as two polypeptides and eventually produce PL^pro^, M^pro^ (also known as 3CLpro), and RdRp. Initial processing of the two polypeptides is by host proteases and then is propagated by PL^pro^ and M^pro^. The viral RdRp is also responsible for the replication and amplification of the viral genome. The viral RNA and the N structural protein are biosynthesized in the host cell cytoplasm, whereas viral structural proteins S, M, and E are biosynthesized in the endoplasmic reticulum and transported to the Golgi apparatus. The viral RNA–N complex and S, M, and E proteins are assembled in ERGIC. The mature virus is produced by the budding process. The virus is then released by exocytosis. Note: The virus enters via membrane fusion in the endo/lyso-somes which requires proteolytic activation by cathepsins (**a**) or via fusion at the cell membrane which requires proteolytic activation by TMPRSS2 (**b**). Furin may also contribute to the entry of the virus, yet its site of action is not fully established. gRNA means genomic RNA and sgRNA means subgenomic RNA.

The life cycle of the virus includes early-stage events and later-stage events (Figure 1a,b). In the first stage, the virus utilizes its spike (S) protein to bind to angiotensin converting enzyme 2 (ACE2) on the host cell membrane [13,14]. The virus enters the host cell after the spike S protein-ACE2 complex is proteolytically activated by transmembrane protease serine 2 (TMPRSS2) (see (b) in Figure 1), which eventually permits the virus-host cell fusion and the release of the viral RNA genome [15]. Alternatively, the bound virus spike S protein can also be proteolytically activated by furin [16]. Further processing is promoted by cathepsins in (endo)lysosomes to ultimately aid in the viral envelope fusion with the host membranes and the release of the viral genome (see (a) in Figure 1) [17].

The RNA genome of SARS-CoV-2 has more than 29,800 nucleotides which encode for about 29 proteins: nonstructural proteins (NSPs; 16 proteins), structural proteins (4 proteins), and accessory proteins (9 proteins) [18,19]. The structural proteins are spike S protein, envelope (E) and membrane (M) proteins which form the viral envelope, and nucleocapsid (N) protein which binds to the virus RNA genome. In the post-entry phase of the virus life cycle (Figure 1), the NSPs domain is expressed as two polypeptides which, after processing, produce papain like protease (PL^pro^) (NSP3), main protease (M^pro^) (also known as 3-chymotrypsin-like protease (3CLpro); NSP5) [20], and RNA-dependent RNA polymerase (RdRp; NSP12) [21]. Initial processing of the two polypeptides is promoted by host proteases, and then, is propagated by the action of the viral PL^pro^ and M^pro^. The viral RdRp is also responsible for the replication and amplification of the viral genome. The viral RNA and the N structural protein are biosynthesized in the host cell cytoplasm, whereas other viral structural proteins including S, M, and E are eventually biosynthesized in the endoplasmic reticulum and transported to the Golgi apparatus. The viral RNA–N complex and S, M, and E proteins are then assembled in the endoplasmic reticulum–Golgi intermediate compartment (ERGIC) to produce a mature virus particle. The mature virus is then released from the Golgi apparatus via a budding process and next from the host cells by exocytosis (Figure 1) [12,22,23,24].

Collectively, the goal of antiviral therapeutics is to inhibit one or more events in the life cycle of the virus in order to impede the propagation of infection. Along these lines, any protein or event in the virus life cycle can be considered as a molecular target for anti-COVID-19 drug development efforts. In this review, we describe the antiviral agents that are currently being tested in clinical trials to block and/or inhibit the advanced events of the virus life cycle. Although the majority of the presented antiviral therapeutics target the viral polymerase or the viral proteases, few other therapeutics target other molecular targets (Table 1).

## 2. Viral Polymerase Inhibitors

### 2.1. Remdesivir (Veklury, GS-5734)

**Figure 2:**
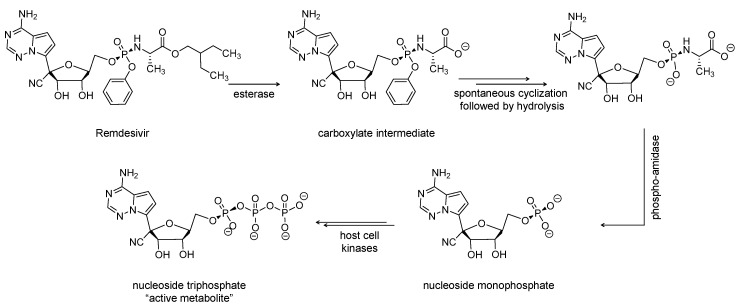
Chemical structure of remdesivir and schematic representation of its metabolic bioactivation. Remdesivir is adenosine monophosphate derivative; it is also classified as nucleotide prodrug. The corresponding triphosphate form is the active form and it is the inhibitor of the viral RNA polymerase.

Remdesivir is an adenosine monophosphate derivative and nucleotide-based antiviral prodrug (Figure 2). Remdesivir received, in May 2020, an emergency use authorization from the U.S. FDA for the treatment of laboratory-confirmed or suspected COVID-19 illness in children and adults hospitalized with severe disease [25]. The parenterally administered drug is being developed by Gilead Sciences, U.S., and has broad-spectrum antiviral activity [26]. It was first studied in 2016 as a potential treatment for Ebola virus [27]. In addition to its activity against SARS-CoV-2, remdesivir has a potential to treat a variety of infections caused by RNA viruses, including SARS-CoV and MERS-CoV [28].

The drug is metabolized to the pharmacologically active nucleoside triphosphate metabolite after being distributed into cells (Figure 2). The triphosphate metabolite acts as a competitive inhibitor of RdRp and thus eventually causes chain elongation termination, which decreases the viral RNA replication [29]. The termination is delayed and happens after the addition of more nucleotides (between 3 and 5). Therefore, remdesivir is described as a direct antiviral agent acting as a delayed chain terminator [30,31]. Importantly, remdesivir avoids proofreading by viral exoribonuclease [28,32]. Currently, remdesivir is being evaluated as a treatment for COVID-19 patients in about 15 studies across the globe. The drug is being tested alone or in combination with merimepodib (NCT04410354; *n* = 40), tocilizumab (NCT04409262; REMDACTA; *n* = 450), or baricitinib (NCT04401579; ACTT2; *n* = 1034). In particular, merimepodib is another antiviral agent that is inhibitor of inosine monophosphate dehydrogenase. The enzyme is required for the synthesis of guanine nucleotides. Merimepodib consequently inhibits the synthesis of DNA and RNA, leading to antiviral and immunosuppressive effects. Thus, remdesivir and merimepodib is a dual-acting antiviral combination with immunosuppressive activity.

Remdesivir itself demonstrated in vitro activity against Vero E6 cells infected with SARS-CoV-2 with an *EC*_50_ value of 0.77 µM (CC_50_ > 100 µM) [33]. Remdesivir also exhibited in vitro activity against SARS-CoV and MERS-CoV in multiple in vitro systems, including primary human airway epithelial cell cultures with sub-micromolar *IC*_50_ values [28]. Remdesivir was also effective against pre-pandemic bat-CoVs, bat-CoVs, and contemporary circulating human coronaviruses in primary human lung cells suggesting a broad-spectrum anti-coronavirus activity. In a mouse model of SARS-CoV, the prophylactic and early therapeutic use of remdesivir significantly decreased the lung viral load and improved the respiratory functions as well as the overall clinical signs of the disease [28]. Furthermore, remdesivir with interferon (INF)-b demonstrated better antiviral activity compared to lopinavir/ritonavir with INF-b in vitro. Compared to lopinavir/ritonavir/INF-b, the prophylactic and therapeutic use of remdesivir also more effectively diminished the pulmonary viral loads and improved the pulmonary function in mice model of MERS-CoV [34]. The efficacy of the prophylactic and therapeutic use of remdesivir was also demonstrated in the rhesus macaque model of MERS-CoV infection [35]. Very recently, remdesivir was also shown to inhibit SARS-CoV-2 replication in human lung cells and primary human airway epithelial cultures (*EC*_50_ = 0.01 μM). In mice infected with a chimeric SARS-CoV encoding RdRp, therapeutic administration of remdesivir diminished lung viral load and improved pulmonary function compared with vehicle-treated mice [36].

As far as clinical trials in humans, a randomized, placebo-controlled, double-blind trial in hospitalized adults (*n* = 236) with severe COVID-19 in China initially revealed that the median time to improvement was not substantially different in the remdesivir group (200 mg on the first day, and then 100 mg/day for 9 days) from that of the placebo group. The mortality rate was also similar in the two groups [37]. Yet, the trial was criticized for being insufficiently powered. Later, a phase 3 randomized, open-label trial in adults (*n* = 397) hospitalized with severe COVID-19 sponsored by Gilead revealed that the time to clinical improvement for 50% of patients was 10 days in the 5-day treatment group relative to 11 days in the 10-day treatment group. The dose regimen used was 200 mg on day 1, followed by 100 mg/day for total of 5 or 10 days. At day 14, about 64.5% of the patients in the 5-day group and 53.8% of the patients in the 10-day group achieved clinical recovery. Patients treated with remdesivir within 10 days of symptoms onset achieved better outcomes relative to those treated after more than 10 days of symptoms [38]. Similar results were obtained in hospitalized adults (*n* = 1600) with moderate COVID-19 (NCT04292730). In an uncontrolled study of hospitalized COVID-19 patients (*n* = 61), most patients needed less oxygen support after receiving remdesivir [39]. Importantly, a phase 3 adaptive, randomized, placebo-controlled study sponsored by the U.S. National Institute of Allergy and Infectious Diseases (NIAID) in hospitalized adults (*n* = 1063) indicated that: (a) the patients in the remdesivir group had shorter median time to recovery (11 days) than the patients in the placebo group (15 days) and (b) remdesivir may decrease the mortality rate from 11.6% in the placebo group to 8% in the treatment group [40]. As of now, the COVID-19 Treatment Guidelines Panel of the U.S. National Institute of Health recommends remdesivir for the treatment of COVID-19 in hospitalized patients with severe disease (requiring supplemental oxygen or on mechanical ventilation or extracorporeal membrane oxygenation). The Panel also indicates that there are no sufficient data to recommend either for or against the use of remdesivir in patients with mild or moderate COVID-19 [41]. Of note, the U.S. FDA warns against the concomitant use of remdesivir and chloroquine or hydroxychloroquine owing to in vitro evidence which suggests that chloroquine blocks the intracellular activation of remdesivir [42]. Moreover, data from the manufacturer’s compassionate use program suggested no safety concerns were identified for remdesivir in pediatric, pregnant, or postpartum patients [43].

### 2.2. Galidesivir (Immucillin-A, BCX4430)

**Figure 3:**
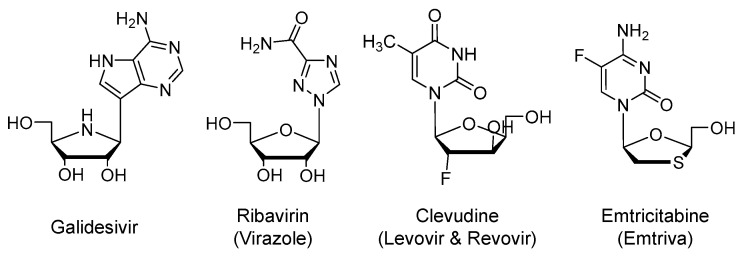
Chemical structures of potential RNA polymerase inhibitors, all of which are nucleoside derivatives that undergo tri-phosphorylation activation to form the corresponding nucleoside triphosphate metabolites as the active forms. Emtricitabine is being tested in COVID-19 patients in combination with tenofovir disoproxil or tenofovir alafenamide. The activation schemes for tenofovir disoproxil or tenofovir alafenamide are provided in the supplementary document.

Galidesivir is an adenosine nucleoside analog (Figure 3) that is an active site inhibitor of RdRp (*EC*_50_ < 50 µM). Similar to remdesivir, it is a prodrug that is metabolized by cellular kinases to the corresponding active form of nucleoside triphosphate. The triphosphate form binds to the active site of the viral enzyme and gets incorporated into the growing viral RNA chain resulting in premature chain termination. The drug is being developed by BioCryst, U.S., and being tested in a phase 1 clinical trial for COVID-19 or Yellow Fever in Brazil in collaboration with the U.S. NIAID (NCT03891420; *n* = 132) [44,45,46,47].

The drug is used parenterally and has demonstrated a broad-spectrum, showing in vitro antiviral activity against at least 20 RNA viruses across eight different virus families including coronaviruses. In animal studies, the drug was effective in protecting against dangerous viruses such as Zika, Yellow Fever, Marburg, and Ebola viruses [44,45,46,47].

### 2.3. Ribavirin (Virazole)

It is an open-ring analog of guanosine nucleoside (Figure 3) that was approved by the U.S. FDA in 1985 for the treatment of respiratory syncytial virus [48]. It is also used systemically for chronic hepatitis C virus (HCV) infection [49] and viral hemorrhagic fever [50]. The drug possesses broad-spectrum antiviral activity against both RNA and DNA viruses. To exert its antiviral activity, the drug is to be activated by phosphorylation to generate the triphosphate nucleotide that acts as an inhibitor of RNA synthesis and viral mRNA capping [51]. Other mechanisms have also been proposed to account for its broad spectrum of antiviral activity. Inhibition of host inosine monophosphate dehydrogenase by ribavirin-monophosphate and the resulting depletion of guanosine triphosphate (GTP) pool has been put forward to be another mechanism of action. Decreased intracellular GTP pool decreases viral protein synthesis and limits the replication of viral genome. Ribavirin is also a mutagen that leads to defective virions [52] and it has immunomodulatory actions [53]. Yet, the drug has a U.S. boxed warning pertaining to the risk of hemolytic anemia and potential complications during pregnancy [54].

Ribavirin is currently being evaluated in few trials for the treatment of COVID-19 patients. It is being tested alone (NCT04356677; *n* = 50) or in combination with nitazoxanide and ivermectin (NCT04392427; *n* = 100) or with lopinavir/ritonavir and INF β-1b (NCT04276688; *n* = 127). Recent computational work has shown that ribavirin binds with high affinity to RdRp of SARS-CoV-2 [55]. Furthermore, the MERS-CoV rhesus macaque model revealed promising results for ribavirin and IFN-α 2b [56]. Nevertheless, mixed results came out of treating MERS-CoV infections with a combination of ribavirin and IFNs (IFN-β1 or IFN-α 2a) [57]. Results from the in vitro testing of ribavirin in Vero E6 cells also indicated that the replication and/or the cellular spread of SARS-CoV was not inhibited at concentrations known to inhibit other sensitive viruses [58]. Interestingly, a recent open-label randomized controlled trial (NCT04276688; *n* = 127) indicated that early triple antiviral therapy of INF-β 1b, ribavirin, and lopinavir/ritonavir was safe and superior to lopinavir/ritonavir alone in alleviating the symptoms and shortening the duration of viral shedding and hospitalization in COVID-19 patients with mild to moderate symptoms [59].

### 2.4. Clevudine (Levovir and Revovir)

It a thymidine nucleoside analog (Figure 3) that was approved in Korea for the treatment of hepatitis B virus (HBV) infection [60]. Similar to previous agents, it is a prodrug that requires phosphorylation to form the corresponding active nucleotide, the triphosphate. Mechanistically, the triphosphate active form appears to noncompetitively inhibit the HBV reverse transcriptase protein priming and DNA synthesis [61]. Importantly, although clevudine showed a potent antiviral response, its long-term use for more than a year led to the development of viral resistance and myopathy [60]. The drug is being evaluated in a phase 2 as a treatment for COVID-19 in Korea (NCT04347915; *n* = 60).

### 2.5. Emtricitabine (Emtriva) in Combination with Tenofovir Disoproxil or Tenofovir Alafenamide

Emtricitabine is a cytosine nucleoside analog (Figure 3) that is a competitive inhibitor of human immunodeficiency virus-1 (HIV-1) reverse transcriptase. It is metabolized by cellular kinases-mediated phosphorylation to the triphosphate form. Emtricitabine triphosphate is the active form that blocks the HIV replication by terminating its genetic chain elongation, and thus, it prevents the generation of complementary DNA from the viral RNA and reduces the viral load. The drug was first approved by the U.S. FDA as an orally bioavailable, once-daily antiretroviral drug in 2003. It is now used in combination with other antiretroviral drugs for the treatment of HIV-1 infection [62,63]. Combinations with tenofovir disoproxil fumarate (Truvada), tenofovir alafenamide (Descovy; 2016), rilpivirine, and tenofovir alafenamide (Odefsey; 2016), or bictegravir and tenofovir alafenamide (Biktarvy; 2018) are available.

In particular, tenofovir disoproxil (Viread; 2001) is an adenine-based acyclic nucleotide analog (Figure S1) that, following activation, acts as a competitive inhibitor of reverse transcriptase, and subsequently, it leads to DNA chain elongation termination. Activation of the drug starts with the hydrolysis of the external esters followed by spontaneous release of carbon dioxide and formaldehyde to form the corresponding tenofovir, a nucleoside monophosphate, which subsequently undergoes two phosphorylation steps to form tenofovir diphosphate, the active drug (Figure S1) [64]. It was first approved in 2001 by the U.S. FDA and is prescribed for the oral treatment of HIV-1 and chronic HBV infections [65]. It is also available in many other combinations with emtricitabine, lamivudine (Cimduo; 2018), doravirine and lamivudine (Delstrigo; 2018), and efavirenz and lamivudine (Symfi; 2018). The efficacy of emtricitabine and tenofovir disoproxil as a prophylactic combination against SARS-CoV-2 infection is being evaluated in a large randomized, double-blind, controlled with placebo clinical trial for health care providers exposed to COVID-19 patients (NCT04334928). The two drugs have been reported by a recent computational work as potential inhibitors of RdRp of SARS-CoV-2 [55,66], yet this potential is to be experimentally confirmed.

Likewise, tenofovir alafenamide (Vemlidy; 2016) is an adenine-based acyclic nucleotide analog that, following activation, acts as a competitive inhibitor of reverse transcriptase and DNA chain elongation termination. The activation of the drug is, however, different and it usually takes place in infected cells by a series of bio-transformations similar to those of remdesivir (Figure S2) [67]. The main advantage of the prodrug, relative to the former prodrug, is that it increases the drug’s oral bioavailability, intestinal diffusion, selectivity of targeting the infected cells, and intracellular half-life. It also decreases the potential renal toxicity of the monophosphate intermediate. Tenofovir alafenamide was first approved in 2016 by the U.S. FDA and is prescribed for the oral treatment of HBV infection [68]. It is also available in many other combinations with emtricitabine (Descovy; 2016), bictegravir and emtricitabine (Biktarvy; 2018), emtricitabine and rilpivirine (Odefsey; 2016), and darunavir/cobicistat and emtricitabine (Symtuza; 2018). The efficacy of emtricitabine and tenofovir alafenamide as a prophylactic combination against SARS-CoV-2 infection is being evaluated in a large randomized, double-blind, controlled with placebo clinical trial for health care providers exposed to COVID-19 patients (NCT04405271; *n* = 1378).

### 2.6. Favipiravir (Avigan, T-705)

**Figure 4:**
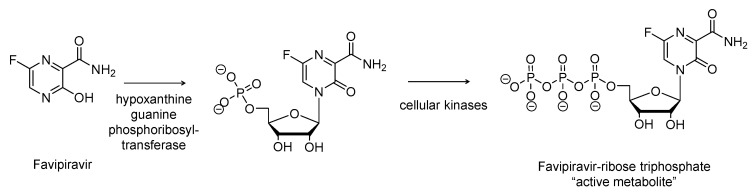
Chemical structure of favipiravir and its metabolic activation. Critical for its activation is the action of hypoxanthine guanine phosphoribosyl-transferase.

Favipiravir was originally developed by Fujifilm group, Japan. It is a pyrazine-carboxamide derivative (Figure 4) with a broad-spectrum antiviral activity. It selectively and potently inhibits the RdRp of RNA viruses [69]. Favipiravir is a prodrug that requires bioactivation in host-infected cells. Its active form is favipiravir-ribose-5′-triphosphate. The first step in the formation of the active species is potentially catalyzed by human hypoxanthine guanine phosphoribosyl-transferase [70], which converts favipiravir into ribose-5′-monophosphate intermediate. The latter intermediate undergoes two phosphorylation steps mediated by the action of host kinases leading to the formation of the ribose-5′-triphosphate active form.

Favipiravir is effective against several strains of influenza viruses, including those that are resistant to existing anti-influenza drugs. Favipiravir also showed an antiviral activity in experimental animals against other RNA viruses, including arenaviruses, alphaviruses, bunyaviruses, and flaviviruses [71]. Furthermore, preliminary results also indicated that favipiravir potentially possesses a moderate activity against Ebola [72]. Importantly, a recent nonrandomized, open-label study in patients (*n* = 80) with non-severe COVID-19 showed that favipiravir (1600 mg orally twice daily on the first day, then 600 mg orally twice daily for thirteen days) with INF-α had significantly better therapeutic effects on SARS-CoV-2 infection, in terms of disease progression and viral clearance, than lopinavir/ritonavir with INF-α [73]. Furthermore, an open-label, prospective, randomized, multicenter study in adults (*n* = 236) with COVID-19 pneumonia in China revealed that favipiravir (1600 mg orally twice daily on the first day, then 600 mg orally twice daily for 7–10 days) was associated with a higher 7-day clinical recovery rate compared to a control group treated with umifenovir, a potential inhibitor of the membrane fusion stage during the virus infection, (200 mg three times daily for 7–10 days). The 7-day clinical recovery rate in patients with moderate COVID-19 pneumonia was 71% in the favipiravir-treated patients, whereas the rate was 56% in the umifenovir-treated patients. Likewise, the 7-day clinical recovery rate in patients with severe to critical COVID-19 pneumonia was 6% versus 0%, respectively [74]. Currently, favipiravir is being studied alone or in combination with tocilizumab, hydroxychloroquine, or oseltamivir for the treatment of COVID-19 in more than 23 clinical trials across the world.

As of now, favipiravir is not available in the U.S. or European countries, perhaps because the animal experiments showed that the antiviral agent can be associated with teratogenic effects. Favipiravir is contraindicated in women with known or suspected pregnancy [75]. Favipiravir is also associated with QT prolongation [76]. It is currently approved to treat novel or re-emerging influenza outbreaks in China and Japan, and it is available as an oral solid dosage form [73,74,76].

### 2.7. AT-527 

It is an investigational, orally active, purine nucleotide prodrug (Figure S3), which has exhibited antiviral activity against many single-stranded, enveloped RNA viruses, including human flaviviruses and coronaviruses [77]. It is a potent inhibitor of viral RdRp [78]. Following oral administration as hemi-sulfate salt, the drug gets converted to the monophosphate form via multiple metabolic activation steps. The first step is catalyzed by the action of human carboxylesterase 1 (CES1) and/or cathepsin A (CatA) to produce the L-alanyl intermediate. Spontaneous hydrolysis followed by histidine triad nucleotide-binding protein 1 (HINT1)-mediated hydrolysis results in the formation of the monophosphate metabolite. Then, the monophosphate is transformed to guanosine analog by adenosine deaminase like protein 1 (ADALP1) and further phosphorylated by guanylate kinase 1 (GUK1) and nucleoside diphosphate kinase (NDPK) to the pharmacologically active form of AT-527 diphosphate (also reported as AT-9010) (Figure S3) [78]. The safety, pharmacokinetics, and antiviral activity of AT-527 was earlier established in HCV-infected subjects with and without cirrhosis [79]. The drug is currently being evaluated in a phase 2 double-blind, randomized, placebo-controlled study to determine its efficacy and safety in patients with moderate COVID-19 symptoms (NCT04396106; *n* = 190).

### 2.8. EIDD-2801 

**Figure 5:**
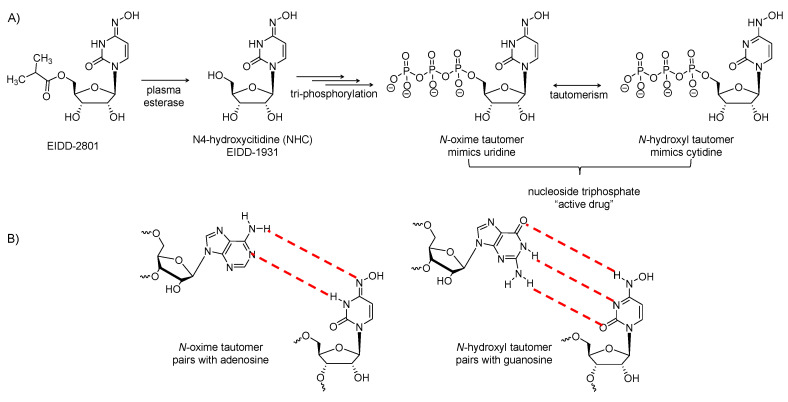
(**A**) Chemical structure of EIDD-2801, an isopropyl ester prodrug of ribonucleoside analog of *β-D-N^4^*-hydroxycytidine. EIDD-2801 is an orally bioavailable nucleoside derivative that is under development for SARS-CoV-2. Also depicted is its activation to the corresponding tri-phosphorylated form which exhibits a broad-spectrum antiviral activity against various RNA viruses, including coronaviruses with resistance mutations to remdesivir. (**B**) The active form exists in two forms: the oxime form which mimics uridine and pairs with adenosine, while the other tautomer mimics cytidine and pairs with guanosine. The drug eventually leads to a viral error catastrophe.

It is the isopropyl-ester prodrug of β-D-*N^4^*-hydroxycytidine (Figure 5A). The prodrug has improved oral bioavailability as it avoids phosphorylation of the *N*^4^-hydroxyl group in the gastrointestinal tract. It is hydrolyzed in vivo to release the parent (EIDD-1931), which distributes into tissues, and upon tri-phosphorylation, it becomes the active triphosphate form. The tri-phosphorylated form has a broad-spectrum antiviral activity against various RNA viruses, including influenza, Ebola, Venezuelan equine encephalitis virus, MERS-CoV, SARS-CoV, SARS-CoV-2 and related zoonotic group 2b or 2c bat coronaviruses [80,81]. It also demonstrated increased potency against a coronavirus with resistance mutations to remdesivir [82]. By the action of RdRp, the active form is incorporated into the genome of RNA viruses, leading to the accumulation of mutations known as viral error catastrophe [80]. The active form exists in two forms (Figure 5B): the oxime form which mimics uridine and pairs with adenosine, while the other tautomer mimics cytidine and pairs with guanosine [81]. In mice infected with MERS-CoV or SARS-CoV, EIDD-2801 administration was found to diminish the virus titer and body weight loss and to improve pulmonary function [80]. Reduced MERS-CoV yields in vitro and in vivo was because of the increase in transition mutation frequency in only the viral RNA. The drug produced similar results in human airway epithelial cells. The drug showed similar results as a prophylactic and as a treatment [80].

The drug was developed at the Emory Institute for Drug Development and it was tested in a phase 1 randomized, double-blind, placebo-controlled, first-in-human study designed to evaluate its safety, tolerability, and pharmacokinetics following oral administration to healthy volunteers (NCT04392219; *n* = 130). It is now being tested in two phase 2 trials in COVID-19 patients (NCT04405570; *n* = 44 and NCT04405739; *n* = 60).

## 3. Viral Protease Inhibitors

### 3.1. Lopinavir/Ritonavir (Kaletra)

**Figure 6:**
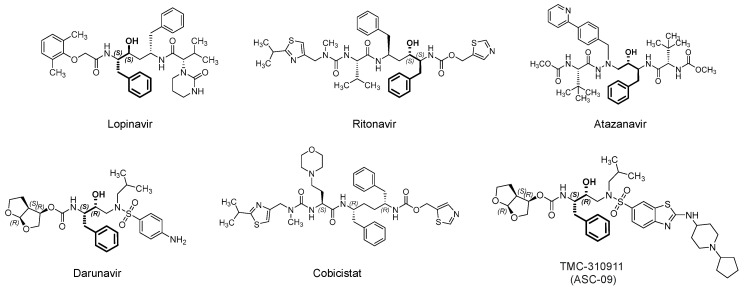
Chemical structures of HIV-1 aspartate protease inhibitors (except cobicistat) that are currently being tested as potential inhibitors of proteases relevant to SARS-CoV-2. Ritonavir and cobicistat are pharmacokinetic boosters. The most frequent tested combination in COVID-19 patients is lopinavir/ritonavir.

Lopinavir (Figure 6) is an orally bioavailable, small peptidomimetic antiretroviral agent that acts as an HIV-1 aspartate competitive inhibitor [83]. The drug inhibits the cleavage of viral Gag-Pol polyprotein precursors into individual functional proteins required for infectious HIV. The inhibition eventually results in the formation of immature, noninfectious viral particles. The drug was approved by the U.S. FDA in combination with ritonavir (Figure 6), which is another antiretroviral aspartate protease inhibitor. Ritonavir does not only provide an additive effective, but it is also a pharmacokinetic booster, i.e., it inhibits the CYP3A-mediated metabolism of lopinavir and thus increases its plasma level [83,84]. Currently, lopinavir/ritonavir is being evaluated for the treatment of SARS-CoV-2 patients in more than 35 interventional trials alone or in conjugation with hydroxychloroquine, inhaled INF-α, INF-β 1b and hydroxychloroquine, or oseltamivir (an inhibitor of neuraminidase in influenza virus) (for details refer to clinicaltrials.gov).

The rationale for using lopinavir is attributed to multiple studies. Lopinavir exhibited an antiviral activity against SARS-CoV-2 virus in Vero E6 cells with an estimated *EC*_50_ value of 26.63 μM [85]. Computational studies have also suggested that lopinavir may inhibit the viral main protease M^pro^, perhaps by targeting its active site [86,87]. Earlier, lopinavir exhibited in vitro activity against SARS-CoV-1 and MERS-CoV [88,89,90]. It also showed beneficial effects in animal studies for the treatment of MERS-CoV [91,92]. Furthermore, there is an evidence of some clinical benefit for lopinavir/ritonavir when used with ribavirin and/or INFs against MERS-CoV and SARS-CoV [88,93,94]. Yet, coronavirus proteases, including M^pro^, do not have a C2-symmetric protein architecture which is the target of lopinavir and all HIV-1 protease inhibitors. This subsequently sheds doubts on the prospect of HIV-1 aspartate protease inhibitors in treating COVID-19. 

In this direction, a randomized, open-label trial in China in COVID-19 patients (*n* = 199), who were hospitalized with severe illness, compared lopinavir/ritonavir (400 mg/100 mg twice a day for 14 days) along with the standard care to the standard care alone [94]. The trial found that the time to achieve clinical improvement was similar in the two groups and that no statistically significant improvement, with respect to the viral load, oxygen therapy duration, hospitalization duration, or time to death, was achieved by the use of the drug combination [95]. Furthermore, a retrospective cohort study in China evaluated the use of lopinavir/ritonavir with or without umifenovir in COVID-19 patients (*n* = 16). On the seventh day, SARS-CoV-2 was not detected in the nasopharyngeal specimens of 35% of lopinavir/ritonavir-treated patients compared to 75% of lopinavir/ritonavir/umifenovir-treated patients. Chest computerized tomography scans were also better in the latter group (29% versus 69%) [96]. Moreover, a randomized, open-label trial in COVID-19 adults (*n* = 127) with mild to moderate symptoms in Hong Kong suggested that adding ribavirin and INF-β-1b to lopinavir/ritonavir increased the efficacy of the treatment when it was initiated within 7 days of the symptoms onset [59]. Other studies also suggested a limited benefit of lopinavir/ritonavir with or without INFs in patients with COVID-19 [97,98,99]. Recently, a small, randomized study in hospitalized COVID-19 adults (*n* = 22) in China compared lopinavir/ritonavir (lopinavir 400 mg/ritonavir 100 mg twice daily for 10 days) against chloroquine (500 mg twice daily for 10 days) and found that chloroquine was linked to a shorter time to RT-PCR conversion and a faster recovery [100].

### 3.2. Darunavir/Cobicistat (Prezcobix)

Darunavir (Figure 6) is another antiretroviral drug that competitively inhibits the HIV-1 aspartate protease [101,102]. In addition to the active site, it has been reported that the flexible darunavir binds to another site on the surface of the enzyme, which accounts for its resilience against potential mutations in the targeted protease [103]. Darunavir was approved in 2015 by the U.S. FDA and usually prescribed in combination with the pharmacokinetic booster cobicistat. Although structurally similar to ritonavir, cobicistat lacks antiviral activity due to the lack of the central phenyl-propanol moiety, a key structural feature of HIV protease inhibitors. 

Darunavir/cobicistat is under clinical investigation for the treatment of SARS-CoV-2 infection. This is potentially attributed to darunavir ability to in vitro inhibit SARS-CoV-2 in Vero E6 cells, albeit at high concentrations (*EC*_50_ = 46.41 µM) [104]. Mechanistically, this could be because darunavir potentially inhibits 3CLpro and/or PL^pro^ of SARS-CoV-2. The two enzymes are important for the viral glycoprotein processing. However, in another in vitro study, darunavir/cobicistat demonstrated no activity against SARS-CoV-2 at clinically relevant concentrations in Caco-2 cells [105]. Furthermore, the results from a randomized controlled trial in China showed that darunavir/cobicistat was not effective in treating COVID-19 patients [106]. Regardless, darunavir is being tried in about three trials in combination with cobicistat (NCT04252274; *n* = 30), ritonavir/hydroxychloroquine (NCT04435587; *n* = 80), ritonavir/oseltamivir, ritonavir/oseltamivir/hydroxychloroquine, or ritonavir/favipiravir/hydroxychloroquine (NCT04303299; *n* = 320). 

TMC310911 (also known as ASC-09) (Figure 6) is structurally similar to darunavir. It is HIV-1 aspartate protease competitive inhibitor with improved antiviral activity. TMC310911 has potent activity against the wild-type HIV-1 and against an extended spectrum of recombinant HIV-1 clinical isolates, including multiple protease inhibitors-resistant strains [107]. Similar to darunavir, it was evaluated with the pharmacokinetic booster ritonavir [108]. Currently, it is being tested in two clinical trials in China in patients infected with SARS-CoV-2. It is being tested in combination with ritonavir (NCT04261907; *n* = 160) or oseltamivir (NCT04261270; *n* = 60). In a recent computational exercise, TMC-310911 was reported as a potential inhibitor of M^pro^ of SARS-CoV-2 [66], yet this potential is to be experimentally confirmed.

### 3.3. Atazanavir (Reyataz)

Atazanavir (Figure 6) is another antiretroviral drug that competitively inhibits the HIV-1 aspartate protease. Atazanavir was approved in 2003 by the U.S. FDA and usually prescribed in combination with the pharmacokinetic booster cobicistat (combination is being marketed under the name Evotaz; 2015). Currently, it is being evaluated alone (NCT04468087; *n* = 189) or in combination with NA-831 (neuroprotective agent; traneurocin) or dexamethasone for the treatment of COVID-19 infection (NCT04452565; *n* = 525) or with nitazoxanide/ritonavir (NCT04459286; *n* = 98). The drug alone or in combination with ritonavir demonstrated in vitro activity against SARS-CoV-2 in Vero E6 cells, human epithelial pulmonary cells (A549), and human monocytes [109,110]. In these studies, atazanavir has been identified as inhibitor of M^pro^. The drug and its combination have been projected to be 10-fold more potent than lopinavir and its combination with ritonavir. The drug also inhibited the virus-induced enhancement of IL-6 and TNF-α levels [109]. In a separate computational study, atazanavir was reported as a potential inhibitor of SARS-CoV-2 helicase, a viral enzyme that unwinds nucleic acids [111].

### 3.4. Danoprevir/Ritonavir 

Danoprevir (Ganovo) is an orally bioavailable 15-membered macrocyclic peptidomimetic antiviral drug (Figure S4). It is an inhibitor of NS3/4A HCV protease, an important processing enzyme complex. It inhibits the protease with an *IC*_50_ value of 0.29 nM [112]. It was approved in China in 2018 to treat chronic HCV patients. At higher concentrations, it also appears to inhibit the aspartate protease of HIV. The NS3/4A protease of HCV is claimed to share a certain level of structural and/or functional similarity to the protease(s) of SARS-CoV-2. Thus, HCV protease inhibitors, including danoprevir, have been proposed as potential therapeutics for COVID-19. This has also been supported by computational work which indicated that HCV protease inhibitors have high binding affinity to 3CLpro of SARS-CoV-2 [113] and by in vitro and clinical studies which showed that patients with SARS-CoV or MERS-CoV may benefit from HCV protease inhibitors [88,90,114]. Currently, danoprevir in combination with ritonavir, is being evaluated in two clinical trials (NCT04345276; *n* = 10) and (with nebulized INF; NCT04291729; *n* = 11) for the treatment of COVID-19 patients. As mentioned earlier, ritonavir is an antiviral and pharmacokinetic booster that extends the systemic exposure of patients to potential therapeutic concentration of danoprevir. In fact, a recent clinical study results under review has indicated that danoprevir/ritonavir combination alleviated the symptoms in COVID-patients and accelerated their recovery in 4–12 days [115].

### 3.5. Maraviroc (Selzentry)

It is a small, synthetic, azabicyclic molecule (Figure S4) that exhibits antiretroviral activity by blocking the interaction between HIV-1 glycoprotein 120 and chemokine receptor 5 (C-C motif receptor 5), on human CD4-presenting cells, that is necessary for HIV-1 to enter cells [116]. The drug was approved by the U.S. FDA in 2007 as an oral treatment for HIV-1. The drug is currently being evaluated in three clinical trials (NCT04441385, NCT04435522, and NCT04475991) for COVID-19 treatment. Recently, it was shown that maraviroc may act as a potential inhibitor of M^pro^ [117]. However, it appears that it is more realistic to assume that the drug is a viral entry inhibitor and potentially acts by blocking the interaction between the viral spike S protein and the host ACE2 receptor [118]. 

## 4. Miscellaneous Antiviral Agents

### 4.1. Selinexor (Xpovio, KPT330) 

**Figure 7:**
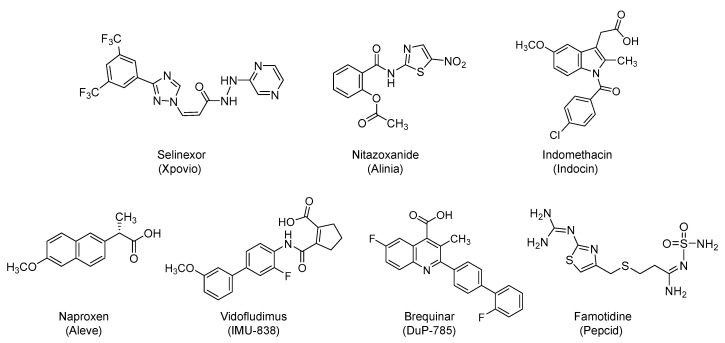
Chemical structures of miscellaneous potential antiviral agents. Of note, the antiviral activity of two NSAIDs (indomethacin and naproxen) and the antiviral activity of dihydroorotate dehydrogenase inhibitors (vidofludimus and brequinar). Many of these drugs also exhibit anti-inflammatory/immunomodulatory effects.

Selinexor is bis(trifluoromethyl)phenyl-triazole-based antineoplastic small molecule (Figure 7). It was first approved in 2019 by the U.S. FDA and is being prescribed with dexamethasone for refractory or relapsed multiple myeloma. Selinexor is an orally bioavailable, selective inhibitor of chromosome region maintenance 1 (CRM1) protein (also known as exportin 1 (XPO1)). CRM1 is the main export factor that shuttles nuclear proteins to the cytoplasm and is typically overexpressed in cancer cells. Its selective inhibition can assist in restoring the endogenous tumor-suppressing processes so as to eliminate cancer cells. Specifically, selinexor selectively and irreversibly modifies the essential Cys528 residue in CRM1, and thus, it blocks CRM1-mediated nuclear export of cargo proteins such as tumor suppressor proteins (p21, p53, pRB, BRCA1/2, FOXO, and others) from the cell nucleus to the cytoplasm. This leads to the accumulation of tumor suppressor proteins in the nucleus. It also results in decreased levels of oncoproteins, cell cycle arrest, and apoptosis of cancer cells without affecting the normal cells [119,120,121]. 

Considering the current viral pandemic, CRM1 has been put forward as a facilitator of the export of viral proteins from the nucleus of the host cell to the cytoplasm as well as an amplifier of the activities of pro-inflammatory transcription factors. Thus, the CRM1 inhibitor selinexor may exert relevant antiviral and anti-inflammatory effects [122,123]. In fact, CRM1 inhibitors have exhibited activity against >20 different viruses, including RNA viruses such as respiratory syncytial virus and influenza virus [122,123]. Furthermore, CRM1 inhibition has also been identified in in vitro assays to have a potential activity against SARS-CoV-2 [124]. CRM1 was found to contribute to exporting several SARS-CoV proteins, such as S, N, 9b, Orf3 and Orf6 out of the nucleus. Thus, CRM1 inhibition is expected to inhibit the viral assembly [125,126,127,128,129]. Moreover, CRM1 has also been found to contribute to the nuclear export and functional inactivation of antioxidant, anti-inflammatory, and cytoprotective transcription factors [130]. High levels of CRM1 are found in multiple inflammatory conditions and may magnify inflammatory responses leading to severe organ damage [131]. In this direction, selinexor and similar inhibitors have exhibited potent anti-inflammatory activity by suppressing the activation of NF_k_B and p38 signaling, leading to reduced cytokines in a variety of models. For example, in a mouse model of sepsis, selinexor increased survival following a lethal dose of endotoxin. Selinexor reduced the inflammatory cytokine secretion of IL-6, TNF-α, and HMGB1 while reducing the numbers of macrophage and polymorphonuclear neutrophils in the mice peritoneal cavity. Selinexor also mitigated lipopolysaccharide-induced lung injury that is similar to acute respiratory distress syndrome [132].

Currently, selinexor is being evaluated in at least two phase 2 randomized trials in the U.S. in COVID-19 patients. One is to evaluate the activity, safety and reduction in mortality of two regimens of low-dose selinexor in patients with moderate or severe COVID-19 (NCT04355676, *n* = 80) and the other is to evaluate the activity of low-dose selinexor and its effect on the clinical recovery, viral load, length of hospitalization, and rate of morbidity and mortality in participants with severe COVID-19 compared to placebo (NCT04349098, *n* = 230). 

### 4.2. Nitazoxanide (Alinia) 

It is a nitro-thiazolyl-salicylamide derivative (Figure 7) with a broad-spectrum antimicrobial activity. The drug is effective against various helminthic, protozoal, bacterial, and viral infections. It was approved by the U.S. FDA in 2002 to be used orally for the treatment of diarrhea caused by *Cryptosporidium parvum* or *Giardia lamblia* [133,134]. It is also off-label used for cryptosporidiosis-associated diarrhea in HIV-infected patients. The drug has been reported as a broad-spectrum antiviral agent that inhibits the replication of several RNA and DNA viruses. Specifically, nitazoxanide has been found to affect influenza A and B viruses as well as neuraminidase inhibitors-resistant influenza viruses. It has also been found to inhibit the replication of coronavirus, dengue virus, HBV, HCV, HIV, Japanese encephalitis virus, norovirus, parainfluenza, rotavirus, respiratory syncytial virus, and yellow fever in cell culture assays [135]. Some of its activity is also potentially attributed to its active metabolite (tizoxanide), desacetyl-nitazoxanide [136]. Currently, nitazoxanide is being studied alone or in combination with hydroxychloroquine, ivermectin, ribavirin/ivermectin, or atazanavir/ritonavir for the prevention/treatment of COVID-19 in about 17 clinical trials (for details refer to clinicaltrials.gov). 

The drug is associated with several mechanisms. The anti-protozoal activity demonstrated by this drug is promoted by the inhibition of pyruvate:ferredoxin oxidoreductase enzyme-dependent electron transfer reaction [137]. However, in the case of influenza, nitazoxanide and its metabolite inhibit the viral hemagglutinin maturation at the post-translational phase with no effect on the M2 protein or on the neuraminidase glycoprotein [138]. Furthermore, nitazoxanide modulates other targets and pathways in vitro including glutamate-gated chloride ion channels and glutathione-S-transferase in nematodes, respiration and other pathways in bacterial and cancer cells, and viral and host transcriptional factors [134]. In fact, nitazoxanide was shown to in vitro inhibit the replication of coronaviruses, including MERS-CoV in cells and the expression of the viral N protein [136,139]. The drug was also reported as a non-competitive inhibitor of thiol oxidoreductase ERp57 and thus demonstrated anti-paramyxovirus activity [140]. Moreover, nitazoxanide was reported to inhibit the production of pro-inflammatory cytokines in peripheral blood mononuclear cells and animal models [136]. In peripheral blood mononuclear cells exposed to influenza virus, nitazoxanide potentiated the release of INF-α and INF-β by fibroblasts [135]. In addition, nitazoxanide appears also to act as a bronchodilator in testing models by blocking the calcium-activated chloride channel TMEM16A [141]. 

### 4.3. NSAIDs: Indomethacin (Indocin) and Naproxen (Aleve)

In one hand, indomethacin is a synthetic, small molecule of *N*-benzoyl-indole-3-acetic acid derivative (Figure 7). It was first approved by the U.S. FDA in 1984 for use as NSAID. It also possesses analgesic and antipyretic properties. It is used for acute mild to moderate pain. It is also used for ankylosing spondylitis, bursitis, tendonitis, osteoarthritis, and rheumatoid arthritis, among other conditions. The known mechanism is the reversible inhibition of cyclooxygenase-1 and 2 enzymes, which leads to decreased formation of prostaglandin precursors [142]. Orally administered indomethacin is being evaluated in combination with hydroxychloroquine and azithromycin in an open-label, single-arm, phase 2 study to determine its efficacy and safety in subjects with mild COVID-19 symptoms (NCT04344457; *n* = 80).

Considering activity against coronaviruses, in vitro studies demonstrated that indomethacin has a potent direct antiviral activity against the SARS coronavirus as determined in monkey Vero E6 cells and human lung epithelial A549 cells as well as against the canine coronavirus as determined in A72 canine cells. Indomethacin blocked the viral RNA synthesis, but not the viral adhesion/entry into the host cells. This effect is independent of the cyclooxygenase inhibition. At a dose rate of 1 mg/kg, indomethacin resulted in more than 1000-fold reduction in the virus yield in coronavirus-infected dogs [143]. Likewise, indomethacin has been reported to be a potent inhibitor of SARS CoV-2 replication in Vero E6 cells with an *IC*_50_ value of 1 µM, which is 10-fold less than its peak plasma concentration, and a selective index of 500-fold. Using a dose of 1 mg/kg in canine coronavirus-infected dogs, indomethacin accelerated the symptoms relieve and saved all infected animals, relative to ribavirin or anti-canine coronavirus serum/canine hemoglobin/canine blood immunoglobulin/INF regimen [144]. Previously, indomethacin also showed antiviral activity against rotavirus infection and vesicular stomatitis virus infection [145,146]. The antiviral activity of indomethacin was proposed to be a result of its binding to peroxisome proliferator activated receptor-γ, aldose reductase, and/or the viral NSP7/NSP8 complex [144]. The inhibition of the NSP7/NSP8 complex has been relatively verified and happened by indomethacin potentially targeting the interface between the host prostaglandin E synthase 2 and the viral NSP7/NSP8. In fact, prostaglandin E synthase 2 itself is inhibited by indomethacin in Vero cells with an *IC*_50_ value of 750 nM [144]. Lastly, in addition to the inhibition of the pro-inflammatory prostaglandin biosynthesis, indomethacin was also found to halt the increase in IL-6 expression caused by lipopolysaccharide-treated U937 cells [147]. Accordingly, the effect of indomethacin on IL-6 may translate into beneficial effect in treating the cytokine storm of COVID-19.

In the other hand, naproxen is another synthetic, small molecule NSAID that is a derivative of 2-naphthalene-acetic acid (Figure 7). It was first approved by the U.S. FDA in 1976 for oral use in the treatment of a host of painful inflammatory conditions such as ankylosing spondylitis, bursitis, polyarticular juvenile idiopathic arthritis, osteoarthritis, rheumatoid arthritis, tendonitis, dysmenorrhea pain, and gout [148,149]. The efficacy of naproxen in the treatment of critically ill, hospitalized COVID-19 patients is being evaluated in a randomized, open label clinical trial (NCT04325633; *n* = 584). 

The drug has been reported to exhibit antiviral activity against influenza A and B viruses with *IC*_50_ values in the low micromolar range. In this arena, naproxen antagonized CRM1-mediated nuclear export of proteins of influenza A and B viruses. Naproxen also provided therapeutic protection to mice infected with influenza B virus [150,151]. In hospitalized patients with influenza, it was found that adding clarithromycin and naproxen to oseltamivir shortened the hospitalization time [150,151]. Naproxen also inhibited the replication of Zika virus by reducing the expression of AXL, the entry cofactor of Zika virus [152]. Naproxen’s antiviral activity against SARS-CoV-2 has also been proposed by a recent computational work and has been attributed its ability to bind to the viral nucleocapsid protein. In fact, it was recently reported that naproxen inhibits SARS-CoV-2 infection in Vero E6 cells and in reconstituted human airway epithelia with *IC*_50_ values comparable to those effective in influenza [153]. Lastly, similar to indomethacin, the anti-inflammatory effects of naproxen may also translate into beneficial effects in treating the cytokine storm of COVID-19.

### 4.4. Vidofludimus Calcium (Immunic AG, IMU-838) and Brequinar (DuP-785)

Vidofludimus is a synthetic small molecule that is under investigation for treating inflammatory bowel disease, multiple sclerosis, and other inflammatory and autoimmune diseases [154,155,156]. It is a biphenyl-carbamoyl-cyclopentene derivative (Figure 7) that is being developed as oral formulation for therapeutic use. Currently, a prospective, randomized, multi-center, double-blinded, and placebo-controlled study is ongoing to evaluate the safety and efficacy of vidofludimus as an adjunct therapy in COVID-19 patients (NCT04379271; *n* = 230) [157].

The investigational drug selectively inhibits dihydroorotate dehydrogenase, an important enzyme for the de novo biosynthesis of pyrimidine-based nucleotides, in activated B and T immune cells. Such inhibition diminishes the pyrimidine pool in these cells, which subsequently exposes the cells to metabolic stress. It also diminishes the release of T helper 1 (Th1) and T helper 17 (Th17) proinflammatory cytokines of IL-17 and IFN-γ, which reduces inflammation [154]. Interestingly, dihydroorotate dehydrogenase inhibition also results in a direct antiviral effect, which has been exhibited in cells infected with hemorrhagic fever-causing viruses, cytomegalovirus, and influenza virus. In fact, IMU-838′s antiviral activity has been demonstrated in vitro against arenavirus, cytomegalovirus, influenza A virus, HCV, and HIV [157]. IMU-838 has also effectively promoted antiviral activity against SARS-CoV-2 [157]. Specifically, IMU-838 inhibited the replication of clinical isolates of SARS-CoV-2. In cellular assays, IMU-838 promoted the anti-SARS-CoV-2 activity at concentrations lower than those that have been considered in previous and ongoing clinical trials [154,155,156,157]. Overall, IMU-838′s antiviral activity against SARS-CoV-2 as well as its selective immunomodulatory effect targeting activated immune cells appear to be interesting, particularly that it potentially prevents the virus reactivation that may happen with other immunomodulatory agents [157].

Likewise, brequinar is a synthetic, small molecule, and quinoline-carboxylic acid derivative (Figure 7) that also inhibits dihydroorotate dehydrogenase. It eventually blocks the de novo biosynthesis of pyrimidine-based nucleotides [158,159]. Accordingly, brequinar possesses immunosuppressive effects. Furthermore, it also possesses antineoplastic properties that can be exploited to enhance the in vivo antitumor activity of other antineoplastic agents [160]. Alternatively, the drug has also antiparasitic effects [160,161]. More importantly, the drug exhibits a broad-spectrum antiviral activity against influenza viruses [162], HIV-1 [163], and foot-and-mouth disease virus [164]. In fact, a pre-print under review has documented the activity of dihydroorotate dehydrogenase inhibitors against RNA viruses, including SARS-CoV-2 [165]. Currently, the drug is being evaluated in a randomized, open-label trial to assess its safety and anti-coronavirus activity in hospitalized adults with COVID-19 (NCT04425252; *n* = 24).

### 4.5. Famotidine (Pepcid) 

Famotidine is a synthetic guandino-thiazole derivative (Figure 7) that acts as a potent and competitive H_2_-blocker. It was first approved by the U.S. FDA in 1986 and now is used as over-the-counter drug. It can be used orally or parenterally to treat gastroesophageal reflux disease, heartburn, and peptic ulcer [166]. Recently, a retrospective non-randomized study by Columbia University, Northwell Health, and Massachusetts General Hospital showed that famotidine, and not proton pump inhibitors, reduced the risk of intubation or death in hospitalized COVID-19 patients (*n* = 84) [167]. Furthermore, a case series indicated that COVID-19 patients (*n* = 10) who frequently self-administered a high-dose of oral famotidine (most frequent dose was 80 mg three times/day for a median of 11 days) reported significant symptoms improvement within 24 hours of starting famotidine [168]. Therefore, intravenously administered famotidine with standard of care (orally administered hydroxychloroquine and has progressed to include remdesivir) is currently being studied in a multi-site, randomized, double-blind, multi-arm historical control, comparative trial that is sponsored by Northwell Health, New York (NCT04370262; *n* = 942). Famotidine has been identified via virtual screening and molecular modeling, docking, and scoring as a potential inhibitor of M^pro^ [169], yet this potential is to be experimentally confirmed.

### 4.6. VERU-111

**Figure 8:**
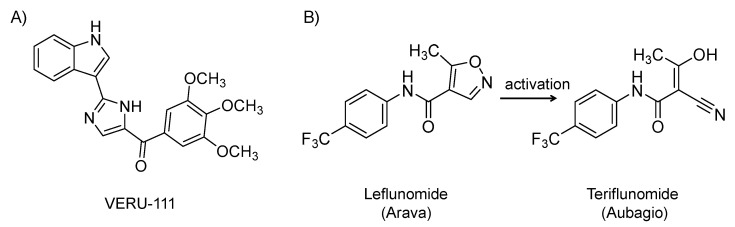
(**A**) The chemical structure of investigational drug VERU-111. The drug exhibits broad antiviral activity because it disrupts the intracellular transport of viruses, including SARS CoV-2, along microtubules, which is critical for viruses to cause infection. It also promotes strong anti-inflammatory effects. (**B**) The chemical structures of leflunomide and its active metabolite teriflunomide. Their potential therapeutic values in COVID-19 patients are attributed to antiviral effects by dihydroorotate dehydrogenase inhibition and to anti-inflammatory effects.

It is an orally bioavailable, 3-substituted indole derivative (Figure 8A) and microtubule depolymerization agent that recognizes colchicine-binding site on tubulin subunits. It has been under clinical development for cancer [170]. Drugs targeting microtubules have broad antiviral activity because they disrupt the intracellular transport of viruses, including SARS CoV-2, along microtubules, which is critical for viruses to cause infection. Microtubule depolymerization agents also have strong anti-inflammatory effects that can be beneficial in mitigating the cytokine storm induced by SARS-CoV-2 infection [171]. The drug is currently being evaluated in a phase 2 randomized, placebo-controlled study for the treatment of SARS-CoV-2 in patients at elevated risk of acute respiratory distress syndrome (NCT04388826; *n* = 40). Of note, it is mechanistically similar to colchicine, which has also been considered in COVID-19 patients [172].

### 4.7. Leflunomide (Arava)

It is a synthetic, small molecule and an isoxazole-carboxamide derivative (Figure 8B). It was approved by the U.S. FDA in 1998 for the oral treatment of rheumatoid arthritis [173,174,175]. It is also used off-label as a replacement therapy in kidney transplant recipients with polyomavirus BK [176] and for cytomegalovirus disease in transplant recipients resistant to standard antivirals [177]. Mechanistically, it is a disease-modifying agent with anti-inflammatory and antiproliferative effects. It inhibits pyrimidine synthesis by inhibiting the mitochondrial enzyme dihydroorotate dehydrogenase, an important enzyme in the de novo synthesis of uridine monophosphate [178]. Leflunomide is a prodrug and the active metabolite (isoxazole ring is open: teriflunomide (Aubagio)) is responsible for its activity [179]. It inhibits rapidly dividing cells such as activated T cells. It has also been found to block the transcription factor NF-κB. It also inhibits tyrosine kinase enzymes. The drug inhibits the replication of cytomegalovirus, herpes simplex virus 1, and polyomavirus BK by interfering with nucleocapsid tegumentation and virion assembly [179]. Given the above activities, a high dose of leflunomide is being evaluated for the treatment of ambulatory patients with mild COVID-19 at the University of Chicago Medicine (NCT04361214; *n* = 20).

### 4.8. Sirolimus (Rapamune)

Sirolimus is a natural macrolide (Figure S5) obtained from *Streptomyces hygroscopicus*. It was first approved by the U.S. FDA in 1999 and is orally used for lymphangioleiomyomatosis and renal transplantation. Mechanistically, the drug is an immunosuppressive agent and an inhibitor of the mammalian target of rapamycin (mTOR). Sirolimus forms an immunosuppressive complex with FK-binding protein-12, and subsequently, the complex inhibits the regulatory kinase, mTOR. This inhibition suppresses cytokine mediated proliferation of T and B cells as well as the antibody production [180,181]. Furthermore, the drug may influence the virus because mTOR complex 1 is involved in the replication of various viruses, including coronavirus [182,183,184]. Furthermore, in vitro studies demonstrated a specific inhibitory activity against MERS-CoV infection by sirolimus [184]. In an open-label, prospective randomized study in H1N1 pneumonia patients, treatment with sirolimus and corticosteroids combination for two weeks alleviated hypoxia, shortened the mechanical ventilation duration, and improved multi-organ function [185]. Accordingly, sirolimus is being tested for the treatment of COVID-19 patients either alone or in combination with hydroxychloroquine in several clinical trials. Likewise, RTB101 (dactolisib), another mTOR inhibitor and phosphoinositide 3-kinase inhibitor, is also being tested in COVID-19 patients (NCT04409327; *n* = 550).

### 4.9. Plitidepsin (Aplidin) 

It is a cyclic depsipeptide natural product (Figure S5) that is extracted from *Aplidium albicans* [186]. It is used for acute lymphoblastic leukemia and, with dexamethasone, for patients with refractory and relapsed multiple myeloma [187]. Recently, the molecule was reported to have in vitro nanomolar potency against the human coronavirus 229E [188]. The mechanism is thought to be similar to its anticancer activity, i.e., targeting eukaryotic translation elongation factor 1-α1, which has been reported to play an unexpected role in the replication and pathogenesis of various RNA viruses [189,190]. The drug is being tested in phase 1 trial in patients with COVID-19 (NCT04382066; *n* = 27).

### 4.10. Cyclosporine A (Gengraf) 

Cyclosporine A is a cyclic peptide that is naturally obtained from the fungus *Beauveria nivea* [191]. It is immunosuppressant and calcineurin inhibitor that inhibits T cell activation [192]. It binds to the intracellular receptor cyclophilin-1 producing a cyclosporine-cyclophilin complex. This complex subsequently inhibits calcineurin, which stops the activation of the nuclear factor of activated T cells (NF-AT) that normally causes inflammatory reactions. The inhibition of NF-AT also leads to lower levels of other factors associated with T helper cell function and thymocyte development. Cyclosporine was first approved by the U.S. FDA in 1983 and is orally, parenterally, or topically used to prevent organ transplant rejection, treat/prevent graft-versus-host disease, and treat various inflammatory and autoimmune conditions such as severe rheumatoid arthritis and psoriasis [191,192,193,194,195]. 

Importantly, low micromolar concentrations of cyclosporin A (<35 µM) substantially impacted the replication of SARS-CoV, human coronavirus 229E, and mouse hepatitis virus in cell culture. Cyclosporin A significantly inhibited gene expression and reduced progeny titers. Cyclosporin A treatment completely blocked SARS-CoV RNA and protein synthesis [196]. Cyclosporine A also in vitro reduced the replication of MERS-CoV, transmissible gastroenteritis coronavirus, porcine epidemic diarrhea virus, and feline coronavirus [196]. In fact, cyclosporine A has demonstrated broad-spectrum antiviral effects. It inhibited the replication of HBV, HCV, and HIV [197]. Cyclosporine also inhibited the replication of Zika virus, West Nile virus, Rift Valley fever virus, and influenza A virus by blocking the interaction of cellular cyclophilins with viral proteins as well as by inhibiting the RNA synthesis [198]. By targeting cyclophilins, the drug can also prevent acute lung injury [199]. Currently, the use of cyclosporin A is being tested in patients with moderate COVID-19 (NCT04412785; *n* = 20), hospitalized COVID-19 patients (NCT04392531; *n* = 120), and combined with topical corticosteroid in COVID-19 patients with acute keratoconjunctivitis (NCT04451239; *n* = 12). It has also been used in psoriatic COVID-19 patients [200].

### 4.11. Deferoxamine (Desferal)

Deferoxamine (Figure S5) is a natural chelating agent from *Streptomyces pilosus*. It was first approved by the U.S. FDA in 1968 and has been parenterally used to treat iron toxicity. The drug complexes with the ferric ion, primarily in the vascular space, to form ferrioxamine complex that gets subsequently eliminated in urine [201,202].

There are reports about unusually high serum ferritin in COVID-19 patients [203]. A retrospective, multicenter cohort study reported increased levels of serum ferritin in COVID-19 non-survivors compared with COVID-19 survivors [99]. The increase in serum ferritin indicates dysregulated iron homeostasis pertaining to oxidative stress and inflammatory response. Dysregulated iron homeostasis may propagate the viral infections leading to severe respiratory illnesses such as pulmonary fibrosis and acute respiratory distress syndrome [204,205,206,207]. Accordingly, the use of iron chelators, particularly deferoxamine, in managing/treating COVID-19 has been proposed [208]. Deferoxamine may exert an antiviral effect by depleting iron availability, which has been shown to play a critical role in the replication of RNA viruses such as HCV, HIV, and West Nile virus [209,210,211,212,213]. In addition to its effect on iron level, deferoxamine also appears to have immunomodulatory effects [209]. Along these lines, deferoxamine mitigated the symptoms of Enterovirus-infected mice and decreased the mortality rate. It also upregulated the B cell levels and improved the neutralizing antibody titer [209]. Deferoxamine has also been reported to in vitro block endothelial inflammation induced by influenza A infection by inhibiting IL-6 synthesis [214]. Deferoxamine may also have antifibrotic effects. An intranasal treatment with deferoxamine was reported to prevent pulmonary fibrosis and pulmonary functional decline in bleomycin-induced pulmonary fibrosis animal model [206].

### 4.12. Atovaquone (Mepron)

Atovaquone is an orally active, synthetic hydroxy-naphthoquinone derivative (Figure S6) with antiparasitic activity. It was approved by the U.S. FDA in 1992 against *Pneumocystis jirovecii* pneumonia [215]. It has also been used to prevent and/or treat toxoplasmosis, malaria, and babesiosis [216,217,218,219]. Mechanistically, it inhibits the electron transport chain in mitochondria, leading to the inhibition of critical metabolic enzymes important for the synthesis of nucleic acids and ATP [220]. It is being evaluated in the U.S. alone (NCT04456153; *n* = 60) or in combination with azithromycin in an open-label, non-randomized study in COVID-19 patients at HonorHealth Clinical Research Institute, U.S. (NCT04339426; *n* = 25). One potential mechanism of action for atovaquone pertaining to SARS-CoV-2 is the inhibition of M^pro^ [221]. Another computational study suggested that it may inhibit RdRp [169]. Importantly, these computational studies are yet to be experimentally confirmed.

### 4.13. Levamisole

It is a tetrahydro-imidazothiazole derivative (Figure S6) that has been used for its immunomodulatory properties. It was first introduced by Janssen in 1969. Ever since, it was used as a disease-modifying drug for the treatment of rheumatoid arthritis [222,223,224]. It was also used with 5-fluorouracil for colon cancer treatment [225]. Currently, it is primarily used as an anthelmintic agent for the treatment of ascariasis and hookworm infections owing to its effect on the parasitic nicotinic acetylcholine receptors [226]. It is also used by pediatric nephrologists as a steroid-sparing agent in childhood steroid-dependent nephrotic syndrome [227].

A recent computational exercise has suggested that levamisole potentially binds to the catalytic domain of the viral PL^pro^, a viral enzyme that processes the newly biosynthesized viral polyproteins and eventually contributes to the maturation and infectivity of the virus [228]. Yet, no experimental validation has been reported thus far. As of now, orally administered levamisole is being studied alone or in combination with budesonide/formoterol inhaler for the treatment of COVID-19 in few clinical trials. The rationale for this use has been because of its immunomodulatory effects. Levamisole acts as an immune-modulator and immune-enhancer by increasing T-cell lymphocyte function and macrophage chemotaxis. It has also been shown to up-regulate toll-like receptors, stimulate neutrophil chemotaxis, and enhance dendritic cell maturation [229,230]. Furthermore, levamisole attenuated alveolar macrophage dysfunction in respiratory virus-infected calves [231].

### 4.14. BLD-2660

It an orally bioavailable, small molecule, reversible covalent inhibitor of calpains, non-lysosomal cysteine proteases, with anti-fibrotic activity. BLD-2660 exhibited potent and selective inhibition of calpains 1,2, and 9 and thus was found to be efficacious in multiple animal fibrosis models of skin, liver, and lung. It exhibited high selectivity against related cysteine proteases. Earlier, several calpain inhibitors were found to reduce the viral replication of SARS-CoV-1 [232,233]. The antiviral activity by calpain inhibition was also verified using siRNA to selectively silence expression of the calpain 2 isoform in SARS-CoV infected cells [232,233]. Undisclosed inhibitors by the developing company were found to reduce SARS-CoV-2 replication in Vero cell-based assays. Furthermore, in preclinical animal models of lung injury and fibrosis, BLD-2660 was shown to reduce the expression/production of IL-6 in injured lungs and to reduce fibrosis [234]. Owing to the antiviral and anti-inflammatory activities of BLD-2660, it has been advanced to a phase 2, randomized, double-blind, placebo-controlled study to evaluate its safety and antiviral activity in COVID-19 hospitalized subjects (NCT04334460; *n* = 120).

### 4.15. N-Acetylcysteine (Acetadote)

It is a small molecule in which the amino group of cysteine amino acid is acetylated (Figure S6). *N*-acetylcysteine was approved by the U.S. FDA in 1963 for the oral use as mucolytic agent and parenteral use to treat acetaminophen overdose [235]. Currently, there are about six ongoing clinical trials to evaluate its use in COVID-19 patients. The rationale for these studies is attributed to its potential antiviral, antioxidant, and immunomodulatory effects. In this arena, several experiments have shown that acetylcysteine may eventually inhibit the viral replication of influenza A [236,237,238]. Acetylcysteine has also been evaluated for use in the treatment of HIV in two randomized studies [239]. Although its effect on the viral load was not consistent, the two studies consistently demonstrated that acetylcysteine substantially increased immunological functions and plasma albumin concentrations [239]. Furthermore, its free sulfhydryl group cleaves the disulfide bonds in mucoproteins which lowers mucous viscosity in patients with cystic fibrosis or chronic obstructive pulmonary disease [240,241,242]. At higher doses, acetylcysteine can also be used as an antioxidant to mitigate the symptoms of many diseases complicated by oxidative stress. In fact, as a component in the synthesis of the antioxidant glutathione, it may reduce the formation of proinflammatory cytokines [243,244]. Acetylcysteine has also been found to mitigate the oxidative stress and improve the inflammatory response in patients with community acquired pneumonia [245].

### 4.16. Artesunate

It is a polycyclic natural product with endoperoxide moiety (Figure S6). It is parenterally used to treat malaria. Initially, there was not enough information about its efficacy against SARS-CoV, except the results of herbal extract screening [246]. It is now being evaluated in few clinical trials alone (NCT04387240; *n* = 22) or in combination with pyronaridine, another antimalarial drug (NCT04475107; *n* = 76 and NCT04532931; *n* = 250). In particular, the combination exhibited antiviral activity against SARS-CoV-2 and influenza viruses in human lung epithelial Calu-3 cells [247]. Other artemisinin-based combinations that appear to be on the rise for potentially treating COVID-19 are mefloquine-artesunate [248] and arteannuin B and lumefantrine [249].

### 4.17. Povidone-Iodine Solution (Betadine)

Povidone-iodine is a chemical complex of polyvinylpyrrolidone polymer and iodine (PVP-I). It is being tested in the U.S., France, United Kingdom, Singapore, and Malaysia as a gargle, mouthwash, or nasal spray to reduce nasopharyngeal viral load in COVID-19 patients. Reports have highlighted the virucidal effect of diluted PVP-I concentration (<10%), without toxic effects on respiratory cilia, olfactory function, or mucosal appearance [250]. In this direction, in vitro studies of 0.23% PVP-I mouthwash suggested that, following a 15-s exposure, the solution can provide effective antiviral activity against influenza virus A (H1N1), MERS-CoV, rotavirus, and SARS-CoV as well as antibacterial activity against *Klebsiella pneumoniae* and *Streptococcus pneumoniae* [251].

### 4.18. Chlorhexidine (Peridex) 

Chlorhexidine is a broad-spectrum, guanidino-containing antimicrobial agent (Figure S6) that is widely used as antiseptic. It was first approved in the U.S. in 1986 and is used for gingivitis and periodontitis. It is also used for the oropharyngeal sanitization to reduce the risk of ventilator-associated or hospital-acquired pneumonia. It is used as a skin disinfectant for preoperative skin preparation. It can also be used to disinfect hands, surface wounds, surgical tools, and surgical scrub, for both patients and healthcare workers [252,253,254,255]. 

Mechanistically, it has both micro-biostatic and micro-biocidal effects depending on its concentration. The former effect happens at low concentrations due to the binding of this cationic molecule to the negatively charged extracellular components of some microbes, which causes an alteration of osmotic equilibrium and leakage of essential ions. At higher concentrations, the molecule gets into the microbial cells and precipitates intracellular components, leading to microbial death [252,253,254,255]. Currently, 0.12% chlorhexidine oral/nasal rinse is being tested in a randomized, open-label, single-institution study so as to evaluate its potential to reduce oro- and naso-pharyngeal viral load in patients with COVID-19 (NCT04344236; *n* = 48). Recently, computational methods have proposed chlorhexidine to be an inhibitor of 3CLpro and/or RdRp of SARS-CoV-2 [169], yet this potential is to be experimentally demonstrated.

### 4.19. Methylene Blue (ProvayBlue) 

Methylene blue is disubstituted phenothiazine derivative (Figure S6) that was approved by the U.S. FDA for the parenteral treatment of methemoglobinemia. It acts by forming leukomethylene blue complex within RBCs. The product is a reducing agent that converts the ferric ion back to the ferrous ion state [256]. Methylene blue has antiviral activity [257,258,259,260,261,262]. A recent study showed that photochemical treatment in conjunction with methylene blue can be used to inactivate SARS-CoV-2 for blood safety and in convalescent plasma therapy [257]. Methylene blue is also known to kill coronavirus (specifically SARS-CoV-2) and HIV in the blood supply when combined with photo-biomodulation [258,259]. Methylene blue and UV/visible light have also been reported to inactivate different viruses, in plasma and in platelet concentrates, including SARS-CoV, MERS-CoV, Ebola virus, Crimean-Congo hemorrhagic fever virus, and Nipah virus [260,261]. A mice model study also demonstrated the ability of methylene blue plus fluorescent light to inactivate West Nile Virus [262]. It is also effective against septic shock [263]. Furthermore, methylene blue is a potent inhibitor of guanylate cyclase, and thus, it improves the arterial pressure and cardiac function in septic shock [264]. It also improves systemic vascular resistance and mean arterial pressure while decreases vasopressor requirements in septic shock [265]. It also demonstrates anti-inflammatory effects because it can inhibit NLRP3 inflammasome activation [266]. The drug is currently being tested in the context of COVID-19 in a few trials alone (NCT04376788; *n* = 15) or in combination with vitamin C and *N*-acetylcysteine (NCT04370288; *n* = 20). 

### 4.20. Inhaled Nitric Oxide

Nitric oxide is an endogenous signaling molecule that is involved in a host of biological processes. The molecule is produced internally by the action of nitric oxide synthase enzyme that catalyzes its production from L-arginine. The antimicrobial activity of nitric oxide has been described against several protozoal and bacterial pathogens as well as against some viruses (herpes simplex virus 1, neurotropic murine coronavirus, and murine hepatitis virus strain 3) [267,268,269]. The role of nitric oxide in SARS-CoV infection was earlier investigated in Vero E6 cells by using the nitric oxide donor *S*-nitroso-*N*-acetyl-penicillamine. The study revealed that the nitric oxide donor substantially inhibited the replication of SARS-CoV in a concentration-dependent fashion (0–400 µM). Nitric oxide inhibited the viral protein and RNA synthesis. Importantly, the study demonstrated that nitric oxide produced by inducible nitric oxide synthase inhibited the replication of SARS-CoV [270]. Another study revealed that nitric oxide causes a reduction in the palmitoylation of the viral spike S protein. This subsequently impedes the fusion between the viral spike S protein and the host ACE2. Moreover, nitric oxide also leads to a substantial decrease in viral RNA production in the early steps of viral replication [271]. These outcomes were attributed to its potential effect on the cysteine proteases of SARS-CoV [272]. Given that the genome of SARS-CoV is significantly similar to SARS-CoV-2, the inhaled nitric oxide therapy in COVID-19 patients is predicted to produce the same effects [273].

Nitric oxide is also a vasodilator; thus, it has been found to promote a selective bronchodilatory effect, which may also benefit COVID-19 patients. The vasodilation is mediated by activating guanylyl cyclase. In fact, the administration of inhaled nitric oxide to critically ill patients of SARS-CoV was found to reverse pulmonary hypertension and to improve severe hypoxia. Nitric oxide administration also shortened the time needed of ventilation support [274]. Along these lines, a positive result was recently reported for inhaled nitric oxide in an outpatient with COVID-19 infection and vasoreactive idiopathic pulmonary arterial hypertension [275].

Currently, there are about 17 clinical trials being performed to evaluate the use of inhaled nitric oxide in COVID-19 patients. Other forms to indirectly deliver nitric oxide include the intravenous administration of nitrite oxide (NCT04401527; *n* = 200) or the nasal delivery of GLS-1200 which contains quinine diluted in saline and has been reported to stimulate nasal cells to produce nitric oxide (NCT04408183; *n* = 225).

### 4.21. Poly-Alcohols: Resveratrol and Quercetin

**Figure 9:**
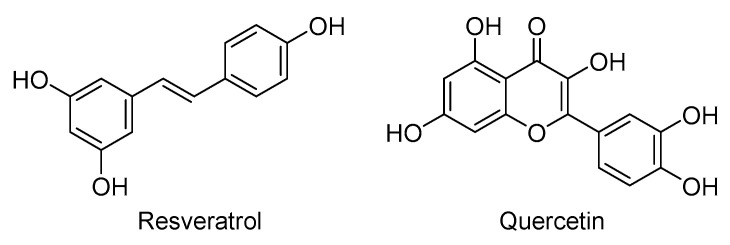
Chemical structures of two naturally occurring polyphenols, i.e., resveratrol and quercetin with a host of beneficial biological effects including antiviral, anti-inflammatory, and antioxidant activities.

On the one hand, resveratrol is a natural polyphenolic stilbene derivative (Figure 9). Resveratrol exhibits several biological activities, including antioxidant, antitumor, anti-inflammatory, oxygen scavenging, and antiviral activities. Resveratrol inhibits TNF-induced activation of NF-κB in a dose-dependent manner. Furthermore, it inhibits cyclooxygenase and hydro-peroxidase enzymes. Not only that but it also inhibits vascular cell adhesion molecule expression and vascular smooth muscle cell proliferation. It also stimulates endothelial nitric oxide synthase activity and inhibits platelet aggregation and LDL peroxidation [276,277,278]. All these effects are expected to help fight COVID-19 infection and its complications. Importantly, resveratrol significantly inhibited MERS-CoV replication in vitro mainly by inhibiting the RNA production [279]. Resveratrol also inhibited the replication of duck enteritis virus [280] and pseudorabies virus [281]. Accordingly, the safety and efficacy of resveratrol in COVID-19 patients are being tested in a randomized double-blind, placebo-controlled proof-of-concept trial (NCT04400890; *n* = 200).

On the other hand, quercetin is a natural penta-hydroxylated flavonoid (Figure 9). Similar to resveratrol, quercetin possesses antioxidant, oxygen scavenging, anti-inflammatory, and cardioprotective effects. Quercetin also inhibits platelet aggregation and lipid peroxidation and affects the function of several kinases [282]. Importantly, quercetin also exhibits a wide spectrum antiviral activity against DNA and RNA viruses. For example, quercetin inhibited several respiratory viruses in cultured cells. It also inhibited the cytopathic effects of rhinoviruses, echoviruses, coxsackieviruses, and polioviruses [283,284,285]. Quercetin also significantly reduced plaque formation by polio virus, herpes simplex virus 1, respiratory syncytial virus, and parainfluenza virus [286]. Furthermore, quercetin inhibited the replication of cytomegalovirus and dengue virus 2 [287]. The antiviral effects of quercetin are thought to be because it either blocks the virus entry or inhibits the viral replication enzymes, i.e., viral polymerases [288]. Other flavonoids have recently been reported as potential inhibitors of M^pro^ [289]. Accordingly, the effect of quercetin alone as a prophylactic or as a treatment is being tested in COVID-19 patients (NCT04377789; 50). It is also being tested with zinc, bromelain, and vitamin C (NCT04468139; *n* = 60).

### 4.22. Macromolecules: Thymalfasin, Lactoferrin, TY027, and XAV-19

Thymalfasin (thymosin-α1, Zadaxin) is a 28-amino acid synthetic peptide that is identical to the natural thymosin-α1 produced by the thymus gland [290]. It possesses immunoregulatory properties owing primarily to its ability to activate various immune cells, particularly T cells. It is used alone or in combination with INFs as an immunomodulator for the treatment of chronic HBV and HCV infections [291,292]. Thymalfasin can also be used for the treatment of chemotherapy-induced immune suppression, and to increase the efficacy of HBV and influenza virus vaccines [293]. Currently, it is being tested in few clinical trials in the context of COVID-19. An important study is the trial to prevent COVID-19 infection in elderly renal dialysis patients (NCT04428008; *n* = 240).

Lactoferrin is another macromolecule that is being considered in the context of COVID-19. It is an iron-binding, multifunctional globular glycoprotein that is widely present in secretory biological fluids, particularly milk [294]. Lactoferrin exhibits a broad-spectrum antimicrobial activity against bacterial, viral, and fungal infections. It also appears to exhibit a clinically relevant activity against some forms of cancer [295], cystic fibrosis [296], and necrotizing enterocolitis [297]. Literature indicates that lactoferrin exhibits activity against a wide range of RNA and DNA viruses, including cytomegalovirus, herpes simplex viruses, HIV, HCV, poliovirus, hantaviruses, rotaviruses, human respiratory syncytial virus, and others [298,299]. Importantly, lactoferrin also appears to exhibit activity against SARS-CoV. It was shown that lactoferrin prevented SARS-CoV from entering human cultured cells. Lactoferrin appeared to block the interaction between the viral spike S protein and heparan sulfate proteoglycans, which serve as an anchoring site on the host cell surface during the early phase of virus infections [300]. Lactoferrin was also found to support the growth of the gut flora as well as the enterocytes proliferation with direct anti-inflammatory/immunomodulatory effects [301]. Currently, lactoferrin is being tested in few clinical trials for COVID-19, including liposomal lactoferrin in COVID-19 patients with mild-to-moderate disease as well as in asymptomatic COVID-19 patients (NCT04475120; *n* = 60).

Other antiviral macromolecules that are being tested against COVID-19 include TY027, a monoclonal antibody (NCT04429529; *n* = 25), and XAV-19, a heterologous swine glyco-humanized polyclonal antibody raised against the spike S protein of SARS-CoV-2 (NCT04453384; *n* = 368).

Lastly, other strategies are being clinically evaluated to treat COVID-19 patients as they may produce direct or indirect antiviral effects. These include the use of convalescent plasma (hyperimmune plasma) from fully recovered individuals [302], immunoglobulins, INFs (such as INF β-1a, INF β-1b, pegylated INF γ-1a and others) [303,304], RNA virus-based gene vector (such as DeltaRex-G) (NCT04378244; *n* = 18), and mesenchymal stem cells [305]. Furthermore, several anti-inflammatory/immune-modulatory drugs, antithrombotic drugs, and vitamins (vitamins C and D) are also being tested to prevent and/or treat severe COVID-19 cases that may arise because of excessive inflammation and thrombotic complications. Most frequently tested anti-inflammatory/immunomodulatory agents are listed in Table 2. Antithrombotic agents that are most frequently being tested in clinical trials for COVID-19 patients are also listed in Table 3.

## 5. Conclusions

The life cycle of SARS-CoV-2 can be divided into two phases. On the one hand, events prior to the viral RNA replication represent the early stage of the virus life cycle. This stage involves the binding of the virus to the host cell receptor, its membrane fusion with the host cell membrane, clathrin-mediated endocytosis, and the release of the viral genome into the host cytoplasmic environment. On the other hand, events that involve RNA replication and subsequent processes represent the later stage of the virus life cycle. Specifically, it entails the RNA replication process, viral protein synthesis and processing, and viral particle assembly, and mature virus release. Notably, events in the two phases are mediated by several interactions among various viral and host proteins. For example, the viral entry is initially mediated by interactions between the viral spike S protein and the host cell receptor ACE2. Furin, TMPRSS2, and cathepsin L enzymes are also important for the early stage of the viral infection. Importantly, the later stage of the viral life cycle involves a different set of virus–host interactions that are mediated by a different set of enzymes. For example, the virus genetic material replication is catalyzed by RdRp and requires nucleotides that are provided by the host cell and biosynthesized by enzymes such as inosine monophosphate dehydrogenase and dihydroorotate dehydrogenase. Furthermore, the resulting viral proteins require further processing which takes place by the action of by M^pro^ and PL^pro^. 

Importantly, any of the virus or the host proteins and the associated events can serve as a potential drug target for the design and development of anti-COVID-19 therapeutics. We previously reviewed potential anti-COVID-19 therapeutics that target the early stage in the viral life cycle [12]. In this review, we summarized potential therapeutics that interfere with the post-entry events of the viral cycle. Important molecular targets to be considered here are the viral RNA polymerase, the viral processing M^pro^ and PL^pro^ enzymes, and the host dihydroorotate dehydrogenase. In this arena, we described potential therapeutics that are currently listed in clinicaltrials.gov. These include small molecule drugs such as nucleoside-based antivirals (galidesivir, ribavirin, clevudine, emtricitabine, and EIDD-2801), nucleotide-based antivirals, (remdesivir, tenofovir, and AT-527), arylpropanol-based peptidomimetics (lopinavir, ritonavir, and others), NSAIDs (indomethacin and naproxen), inhaled nitric oxide, polyalcohol natural products (resveratrol and quercetin), antiprotozoal and antimalarial drugs (nitazoxanide, levamisole, atovaquone, and artesunate), cyclic and acyclic natural peptides (deferoxamine, plitidepsin, and cyclosporine A), and a few others. The case of favipiravir is also unique as it is a non-nucleoside(tide) drug, yet it gets subsequently activated to the corresponding active form of favipiravir-ribose-triphosphate. The described therapeutics also include macromolecules such as thymalfasin, lactoferrin, TY027, and XAV-19.

Many of the above drugs are currently approved therapeutics for other indications; thus, they present a unique repurposing opportunity. Yet, others are new molecular entities such as AR-527, EIDD-2801, ACS-09, vidofludimus, VERU-111, and BLD-2660. Interestingly, the reviewed therapeutics exploit a range of mechanisms which will essentially enhance the likelihood of obtaining effective therapeutics in a timely manner. Furthermore, many of the presented therapeutics promote pharmacological effects beyond the antiviral effects. Of note are the anti-inflammatory/immune-modulatory effects of selinexor, NSAIDs, VERU-111, leflunomide, and BLD-2660. These effects are of enormous significance due to the confirmed excessive inflammation in the severe cases of COVID-19. It is worth mentioning here that testing of the described therapeutics in COVID-19 clinical trials is based on either initial clinical observations or reported activity against previous outbreaks of SARS-CoV and MERS-CoV.

Lastly, although the individual use of the described therapeutics can be beneficial, yet a combination of the above drugs with each other and/or with those that impact the early stage of the viral life cycle will likely lead to a higher success rate in treating the critically ill patients. The clinical outcome is likely to be further improved by the addition of immune-therapeutics and anticoagulants to address the issues of cytokine storm and coagulopathies, respectively. Based on initial results, remdesivir, favipiravir, EIDD-2801, and selinexor appear to carry the most promising therapeutic effects. In fact, on May 1, 2020, the U.S. FDA issued an emergency use authorization for remdesivir to be distributed and used by licensed health care providers to treat adults and children hospitalized with severe COVID-19 [25]. On May 30, 2020, the Russian Health Ministry approved a generic version of favipiravir named avifavir for the treatment of COVID-19 in the hospital settings [10].

## Figures and Tables

**Table 1:** Potential anti-COVID-19 therapeutics that are being tested in clinical trials based on targeting the post-entry events of the life cycle of SARS-CoV-2 ^a^.

**Viral Polymerase Inhibitors**	
Remdesivir	Emtricitabine & tenofovir alafenamide
Galidesivir	Favipiravir
Ribavirin	AT-527
Clevudine	EIDD-2801
Emtricitabine & tenofovir disoproxil	
**Viral protease inhibitors**	
Lopinavir/ritonavir	Atazanavir
Darunavir/cobicistat	Danoprevir/ritonavir
ASC-09	Maraviroc
**Miscellaneous antiviral agents**	
Selinexor	Levamisole
Nitazoxanide	BLD-2660
NSAIDs (Indomethacin and naproxen) ^b^	N-Acetylcysteine
Vidofludimus	Artesunate
Brequinar	Povidone-iodine solution
Famotidine	Chlorhexidine
VERU-111	Methylene blue
Leflunomide	Inhaled nitric oxide
Sirolimus	Poly-alcohols (Resveratrol & quercetin)
Plitidepsin	Thymalfasin
Cyclosporine	Lactoferrin
Deferoxamine	TY027
Atovaquone	XAV-19

^a^ Some of the listed therapeutics have multiple antiviral mechanisms. Some also have anti-inflammatory effects. ^b^ NSAIDs is an acronym for nonsteroidal anti-inflammatory drugs.

**Table 2:** Most frequently evaluated anti-inflammatory/immunomodulatory drugs in clinical trials for COVID-19 ^a^.

Therapy	Type	Mechanism	No. of Interventional Clinical Trials ^b^
Tocilizumab (Actemra)	Humanized monoclonal antibody	IL-6 receptor blocker	~41
Ruxolitinib (Jakafi)	Small molecule	JAK 1 and 2 inhibitor	~15
Colchicine (Colcrys)	Small molecule	Inhibition of NLRP3 inflammasome and of microtubule polymerization	~15
Anakinra (Kineret)	Recombinant non-glycosylated human polypeptide	IL-1 receptor antagonist	~13
Methylprednisolone (DEPOMedrol)	Small molecule	Intracellular receptor-mediated gene expression & suppression of migration of polymorphonuclear leukocytes, among others	~13
Baricitinib (Olumiant)	Small molecule	JAK 1 and 2 inhibitor	~12
Sarilumab (Kevzara)	Human monoclonal antibody	IL-6 receptor blocker	~10
Dexamethasone (Decadron, Active Injection D)	Small molecule	Stimulation of glucocorticoid receptors, suppression of neutrophil migration, suppression of inflammatory mediators production, and reversal of increased capillary permeability	~10
Sirolimus (Rapamune)	Small molecule	mTOR pathway inhibitor	~4
Mavrilimumab	Human monoclonal antibody	GM-CSF receptor blocker	~4
Siltuximab (Sylvant)	Chimeric monoclonal antibody	Anti-IL-6	~2
Canakinumab (Ilaris)	Human monoclonal antibody	Anti-IL-1β	~2

^a^ From clinicaltrials.gov, ^b^ No. is as of July 25, 2020. Abbreviations: IL: Interleukin; JAK: Janus kinase; NLRP3: NOD-like receptor protein 3; mTOR: mammalian target of rapamycin; GM-CSF: granulocyte macrophage colony-stimulating factor.

**Table 3:** Most frequently evaluated antithrombotics in clinical trials for COVID-19 ^a^.

Therapy	Type	Mechanism	No. of Interventional Clinical Trials ^b^
Unfractionated heparin or Low molecular weight heparins (Enoxaparin, Dalteparin, Tinzaparin, and others)	Sulfated glycosaminoglycans	Activation of antithrombin to inhibit thrombin and factor Xa	~23
Aspirin (Acetylsalicylic acid)	Small molecule	Cyclo-oxygenase inhibitor	~8
Rivaroxaban (Xarelto)	Small molecule	Direct factor Xa inhibitor	~5
Dipyridamole (Aggrenox)	Small molecule	Nucleoside transport inhibitor and a phosphodiesterase 3 inhibitor	~3
Clopidogrel (Plavix)	Small molecule	Irreversible adenosine diphosphate receptor blocker	~3

^a^ From clinicaltrials.gov, ^b^ No. is as of 25 July 2020.

## Supplementary Materials

The following are available online at https://www.mdpi.com/1999-4915/12/10/1092/s1, Figure S1: The chemical structure of tenofovir disoproxil and schematic representation of its metabolic bioactivation, Figure S2: The chemical structure of tenofovir alafenamide and schematic representation of its metabolic bioactivation, Figure S3: The chemical structure of AT-527 and schematic representation of its metabolic bioactivation, Figure S4: The chemical structure of danoprevir and maraviroc, Figure S5: The chemical structures of three natural products that are currently being tested against COVID-19, Figure S6: The chemical structures of various agents with potential antiviral activity attributed to various mechanisms.

## Abbreviations

3CLpro	3-Chymotrypsin-like protease
ACE2	Angiotensin converting enzyme 2
ADALP1	Adenosine deaminase like protein 1
CES1	Carboxylesterase 1
COVID-19	Coronavirus infectious disease of 2019
CRM1	Chromosome region maintenance 1
ERGIC	Endoplasmic reticulum–Golgi intermediate compartment
GTP	Guanosine triphosphate
GUK1	Guanylate kinase 1
HINT1	Histidine triad nucleotide-binding protein 1
HBV	Hepatitis B virus
HCV	Hepatitis C virus
HIV	Human immunodeficiency virus
INF	Interferon
IL	Interleukin
MERS-CoV	Middle East respiratory syndrome coronavirus
M^pro^	Main protease
NDPK	Nucleoside diphosphate kinase
NSAIDs	Nonsteroidal anti-inflammatory drugs
NSP	Nonstructural protein
NF-AT	Nuclear factor of activated T-cells
mTOR	Mammalian target of rapamycin
PLpro	Papain-like protease
RdRp	RNA-dependent RNA polymerase
SARS-CoV	Severe acute respiratory syndrome coronavirus
SARS-CoV-2	Severe acute respiratory syndrome coronavirus-2
TRMPSS2	Transmembrane protease serine 2

## References

[1] Peiris J.S., Yuen K.Y., Osterhaus A.D., Stöhr K. (2003). The severe acute respiratory syndrome. N. Engl. J. Med..

[2] De Groot R.J., Baker S.C., Baric R.S., Brown C.S., Drosten C., Enjuanes L., Fouchier R.A., Galiano M., Gorbalenya A.E., Memish Z.A. (2013). Middle East respiratory syndrome coronavirus (MERS-CoV): Announcement of the Coronavirus Study Group. J. Virol..

[3] Coronaviridae Study Group of the International Committee on Taxonomy of Viruses (2020). The species Severe acute respiratory syndrome-related coronavirus: Classifying 2019-nCoV and naming it SARS-CoV-2. Nat. Microbiol..

[4] Dong E., Du H., Gardner L. (2020). An interactive web-based dashboard to track COVID-19 in real time. Lancet Infect. Dis..

[5] Lu L., Zhong W., Bian Z., Li Z., Zhang K., Liang B., Zhong Y., Hu M., Lin L., Liu J. (2020). A comparison of mortality-related risk factors of COVID-19, SARS, and MERS: A systematic review and meta-analysis. J. Infect..

[6] Cucinotta D., Vanelli M. (2020). WHO Declares COVID-19 a Pandemic. Acta Biomed..

[7] Ruch Y., Kaeuffer C., Guffroy A., Lefebvre N., Hansmann Y., Danion F. (2020). Rapid Radiological Worsening and Cytokine Storm Syndrome in COVID-19 Pneumonia. Eur. J. Case. Rep. Intern. Med..

[8] Marietta M., Coluccio V., Luppi M. (2020). COVID-19, coagulopathy and venous thromboembolism: More questions than answers. Intern. Emerg. Med..

[9] National Institute of Health COVID-19 Treatment Guidelines. Antiviral Therapy. https://www.covid19treatmentguidelines.nih.gov/antiviral-therapy/.

[10] Russian Direct Investment Fund. https://rdif.ru/Eng_fullNews/5220/.

[11] National Institute of Health COVID-19 Treatment Guidelines. Corticosteroids. https://www.covid19treatmentguidelines.nih.gov/dexamethasone/.

[12] Al-Horani R.A., Kar S., Aliter K.F. (2020). Potential Anti-COVID-19 Therapeutics that Block the Early Stage of the Viral Life Cycle: Structures, Mechanisms, and Clinical Trials. Int. J. Mol. Sci..

[13] Zhou P., Yang X.-L., Wang X.-G., Hu B., Zhang L., Zhang W., Si H.-R., Zhu Y., Li B., Huang C.-L. (2020). A pneumonia outbreak associated with a new coronavirus of probable bat origin. Nature.

[14] Shang J., Wan Y., Luo C., Ye G., Geng Q., Auerbach A., Li F. (2020). Cell entry mechanisms of SARS-CoV-2. Proc. Natl. Acad. Sci. USA.

[15] Hoffmann M., Kleine-Weber H., Schroeder S., Krüger N., Herrler T., Erichsen S., Schiergens T.S., Herrler G., Wu N.H., Nitsche A. (2020). SARS-CoV-2 Cell Entry Depends on ACE2 and TMPRSS2 and Is Blocked by a Clinically Proven Protease Inhibitor. Cell.

[16] Hoffmann M., Kleine-Weber H., Pöhlmann S.A. (2020). Multibasic cleavage site in the spike protein of SARS-CoV-2 is essential for infection of human lung cells. Mol. Cell.

[17] Liu T., Luo S., Libby P., Shi G.P. (2020). Cathepsin L-selective inhibitors: A potentially promising treatment for COVID-19 patients. Pharmacol. Ther..

[18] Khailany R.A., Safdar M., Ozaslan M. (2020). Genomic characterization of a novel SARS-CoV-2. Gene Rep..

[19] Astuti I., Ysrafil (2020). Severe Acute Respiratory Syndrome Coronavirus 2 (SARS-CoV-2): An overview of viral structure and host response. Diabetes Metab. Syndr. Clin. Res. Rev..

[20] Zhang L., Lin D., Sun X., Curth U., Drosten C., Sauerhering L., Becker S., Rox K., Hilgenfeld R. (2020). Crystal structure of SARS-CoV-2 main protease provides a basis for design of improved α-ketoamide inhibitors. Science.

[21] Gao Y., Yan L., Huang Y., Liu F., Zhao Y., Cao L., Wang T., Sun Q., Ming Z., Zhang L. (2020). Structure of the RNA-dependent RNA polymerase from COVID-19 virus. Science.

[22] Jiang S., Hillyer C., Du L. (2020). Neutralizing antibodies against SARS-CoV-2 and other human coronaviruses. Trends Immunol..

[23] Atri D., Siddiqi H.K., Lang J., Nauffal V., Morrow D.A., Bohula E.A. (2020). COVID-19 for the Cardiologist: A Current Review of the Virology, Clinical Epidemiology, Cardiac and Other Clinical Manifestations and Potential Therapeutic Strategies. JACC: Basic Transl. Sci..

[24] Du L., He Y., Zhou Y., Liu S., Zheng B.-J., Jiang S. (2009). The spike protein of SARS-CoV--a target for vaccine and therapeutic development. Nat. Rev. Microbiol..

[25] The U.S. Food and Drug Administration. https://www.fda.gov/media/137564/download.

[26] Scavone C., Brusco S., Bertini M., Sportiello L., Rafaniello C., Zoccoli A., Berrino L., Racagni G., Rossi F., Capuano A. (2020). Current pharmacological treatments for COVID-19: What’s next?. Br. J. Pharmacol..

[27] Warren T.K., Jordan R., Lo M.K., Ray A.S., Mackman R.L., Soloveva V., Siegel D., Perron M., Bannister R., Hui H.C. (2016). Therapeutic efficacy of the small molecule GS-5734 against Ebola virus in rhesus monkeys. Nature.

[28] Sheahan T.P., Sims A.C., Graham R.L., Menachery V.D., Gralinski L.E., Case J.B., Leist S.R., Pyrc K., Feng J.Y., Trantcheva I. (2017). Broad-spectrum antiviral GS-5734 inhibits both epidemic and zoonotic coronaviruses. Sci. Transl. Med..

[29] Eastman R.T., Roth J.S., Brimacombe K.R., Simeonov A., Shen M., Patnaik S., Hall M.D. (2020). Remdesivir: A Review of Its Discovery and Development Leading to Emergency Use Authorization for Treatment of COVID-19. ACS Cent. Sci..

[30] Gordon C.J., Tchesnokov E.P., Feng J.Y., Porter D.P., Götte M. (2020). The antiviral compound remdesivir potently inhibits RNA-dependent RNA polymerase from Middle East respiratory syndrome coronavirus. J. Biol. Chem..

[31] Gordon C.J., Tchesnokov E.P., Woolner E., Perry J.K., Feng J.Y., Porter D.P., Götte M. (2020). Remdesivir is a direct-acting antiviral that inhibits RNA-dependent RNA polymerase from severe acute respiratory syndrome coronavirus 2 with high potency. J. Biol. Chem..

[32] Ferner. R.E., Aronson J.K. (2020). Remdesivir in covid-19. BMJ.

[33] Wang M., Cao R., Zhang L., Yang X., Liu J., Xu M., Shi Z., Hu Z., Zhong W., Xiao G. (2020). Remdesivir and chloroquine effectively inhibit the recently emerged novel coronavirus (2019-nCoV) in vitro. Cell Res..

[34] Sheahan T.P., Sims A.C., Leist S.R., Schäfer A., Won J., Brown A.J., Montgomery S.A., Hogg A., Babusis D., Clarke M.O. (2020). Comparative therapeutic efficacy of remdesivir and combination lopinavir, ritonavir, and interferon beta against MERS-CoV. Nat. Commun..

[35] De Wit E., Feldmann F., Cronin J., Jordan R., Okumura A., Thomas T., Scott D., Cihlar T., Feldmann H. (2020). Prophylactic and therapeutic remdesivir (GS-5734) treatment in the rhesus macaque model of MERS-CoV infection. Proc. Natl. Acad. Sci. USA.

[36] Pruijssers A.J., George A.S., Schäfer A., Leist S.R., Gralinksi L.E., Dinnon K.H., Yount B.L., Agostini M.L., Stevens L.J., Chappell J.D. (2020). Remdesivir Inhibits SARS-CoV-2 in Human Lung Cells and Chimeric SARS-CoV Expressing the SARS-CoV-2 RNA Polymerase in Mice. Cell Rep..

[37] Wang Y., Zhang D., Du G., Du R., Zhao J., Jin Y., Fu S., Gao L., Cheng Z., Lu Q. (2020). Remdesivir in adults with severe COVID-19: A randomised, double-blind, placebo-controlled, multicentre trial. Lancet.

[38] Goldman J.D., Lye D., Hui D.S., Marks K.M., Bruno R., Montejano R., Spinner C.D., Galli M., Ahn M.-Y., Nahass R.G. (2020). Remdesivir for 5 or 10 Days in Patients with Severe Covid-19. N. Engl. J. Med..

[39] Grein J., Ohmagari N., Shin D., Diaz G., Asperges E., Castagna A., Feldt T., Green G., Green M.L., Lescure F.X. (2020). Compassionate Use of Remdesivir for Patients with Severe Covid-19. N. Engl. J. Med..

[40] The National Institutes of Health (2020). NIH clinical trial shows remdesivir accelerates recovery from advanced COVID-19. https://www.nih.gov/news-events/news-releases/nih-clinical-trial-shows-remdesivir-accelerates-recovery-advanced-covid-19.

[41] The U.S. National Institutes of Health Remdesivir. https://www.covid19treatmentguidelines.nih.gov/antiviral-therapy/remdesivir/.

[42] The U.S. Food and Drug Administration Fact Sheet for Health Care Providers Emergency Use Authorization (EUA) of Veklury^®^ (remdesivir). https://www.fda.gov/media/137566/download.

[43] Gilead Sciences Press Release: Gilead Presents Additional Data on Investigational Antiviral Remdesivir for the Treatment of COVID-19. https://www.gilead.com/news-and-press/press-room/press-releases/2020/7/gilead-presents-additional-data-on-investigational-antiviral-remdesivir-for-the-treatment-of-covid-19.

[44] Warren T.K., Wells J., Panchal R.G., Stuthman K.S., Garza N.L., Van Tongeren S.A., Dong L., Retterer C.J., Eaton B.P., Pegoraro G. (2014). Protection against filovirus diseases by a novel broad-spectrum nucleoside analogue BCX4430. Nature.

[45] Westover J.B., Mathis A., Taylor R., Wandersee L., Bailey K.W., Sefing E.J., Hickerson B.T., Jung K.-H., Sheridan W.P., Gowen B.B. (2018). Galidesivir limits Rift Valley fever virus infection and disease in Syrian golden hamsters. Antivir. Res..

[46] Biocryst Glidesivir. https://www.biocryst.com/our-program/galidesivir/.

[47] Julander J.G., Siddharthan V., Evans J., Taylor R., Tolbert K., Apuli C., Stewart J., Collins P., Gebre M., Neilson S. (2017). Efficacy of the broad-spectrum antiviral compound BCX4430 against Zika virus in cell culture and in a mouse model. Antivir. Res..

[48] Trang T.P., Whalen M., Hilts-Horeczko A., Doernberg S.B., Liu C. (2018). Comparative effectiveness of aerosolized versus oral ribavirin for the treatment of respiratory syncytial virus infections: A single-center retrospective cohort study and review of the literature. Transpl. Infect. Dis..

[49] Dienstag J.L., McHutchison J.G. (2006). American Gastroenterological Association medical position statement on the management of hepatitis C. Gastroenterology.

[50] Ergönül Ö., Keske Ş., Çeldir M.G., Kara I.A., Pshenichnaya N., Abuova G., Blumberg L., Gönen M. (2018). Systematic Review and Meta-analysis of Postexposure Prophylaxis for Crimean-Congo Hemorrhagic Fever Virus among Healthcare Workers. Emerg. Infect. Dis..

[51] Cameron C.E., Castro C. (2001). The mechanism of action of ribavirin: Lethal mutagenesis of RNA virus genomes mediated by the viral RNA-dependent RNA polymerase. Curr. Opin. Infect. Dis..

[52] Te H.S., Randall G., Jensen D.M. (2007). Mechanism of action of ribavirin in the treatment of chronic hepatitis C. Gastroenterol. Hepatol..

[53] Martin P., Jensen D.M. (2008). Ribavirin in the treatment of chronic hepatitis C. J. Gastroenterol. Hepatol..

[54] Sung H., Chang M., Saab S. (2011). Management of hepatitis C antiviral therapy adverse effects. Curr. Hepat. Rep..

[55] Elfiky A.A. (2020). Ribavirin, Remdesivir, Sofosbuvir, Galidesivir, and Tenofovir against SARS-CoV-2 RNA dependent RNA polymerase (RdRp): A molecular docking study. Life Sci..

[56] Falzarano D., De Wit E., Rasmussen A.L., Feldmann F., Okumura A., Scott D.P., Brining D., Bushmaker T., Martellaro C., Baseler L. (2013). Treatment with interferon-α2b and ribavirin improves outcome in MERS-CoV-infected rhesus macaques. Nat. Med..

[57] Arabi Y.M., Mandourah Y., Al-Hameed F., Sindi A.A., Almekhlafi G.A., Hussein M.A., Jose J., Pinto R., Al-Omari A., Kharaba A. (2018). Corticosteroid Therapy for Critically Ill Patients with Middle East Respiratory Syndrome. Am. J. Respir. Crit. Care Med..

[58] Tan E.L., Ooi E.E., Lin C.-Y., Tan H.C., Ling A.E., Lim B., Stanton L.W. (2004). Inhibition of SARS coronavirus infection in vitro with clinically approved antiviral drugs. Emerg. Infect. Dis..

[59] Hung I.F., Lung K.C., Tso E.Y., Liu R., Chung T.W., Chu M.Y., Ng Y.Y., Lo J., Chan J., Tam A.R. (2020). Triple combination of interferon beta-1b, lopinavir-ritonavir, and ribavirin in the treatment of patients admitted to hospital with COVID-19: An open-label, randomised, phase 2 trial. Lancet.

[60] Jang J.-H., Kim J.-W., Jeong S.H., Myung H.-J., Kim H.S., Park Y.S., Lee S.H., Hwang J.-H., Kim N., Lee D.H. (2011). Clevudine for chronic hepatitis B: Antiviral response, predictors of response, and development of myopathy. J. Viral. Hepat..

[61] Jones S.A., Murakami E., Delaney W., Furman P., Hu J. (2013). Noncompetitive inhibition of hepatitis B virus reverse transcriptase protein priming and DNA synthesis by the nucleoside analog clevudine. Antimicrob. Agents Chemother..

[62] The U.S. Food and Drug Administration Prescribing Information for Emtriva. https://www.accessdata.fda.gov/drugsatfda_docs/label/2012/021500s019lbl.pdf.

[63] Frampton J.E., Perry C.M. (2005). Emtricitabine: A review of its use in the management of HIV infection. Drugs.

[64] Murphy R.A., Valentovic M.A. (2017). Factors Contributing to the Antiviral Effectiveness of Tenofovir. J. Pharmacol. Exp. Ther..

[65] Buti M., Tsai N., Petersen J., Flisiak R., Gurel S., Krastev Z., Aguilar Schall R., Flaherty J.F., Martins E.B., Charuworn P. (2015). Seven-year efficacy and safety of treatment with tenofovir disoproxil fumarate for chronic hepatitis B virus infection. Dig. Dis. Sci..

[66] Copertino D.C., Lima B., Duarte R., Wilkin T., Gulick R., De Mulder Rougvie M., Nixon D. (2020). Antiretroviral Drug Activity and Potential for Pre-Exposure Prophylaxis Against COVID-19 and HIV Infection. ChemRxiv.

[67] Birkus G., Bam R.A., Willkom M., Frey C.R., Tsai L., Stray K.M., Yant S.R., Cihlar T. (2015). Intracellular Activation of Tenofovir Alafenamide and the Effect of Viral and Host Protease Inhibitors. Antimicrob. Agents Chemother..

[68] Kaneko S., Kurosaki M., Tamaki N., Itakura J., Hayashi T., Kirino S., Osawa L., Watakabe K., Okada M., Wang W. (2019). Tenofovir alafenamide for hepatitis B virus infection including switching therapy from tenofovir disoproxil fumarate. J. Gastroenterol. Hepatol..

[69] Furuta Y., Komeno T., Nakamura T. (2017). Favipiravir (T-705), a broad-spectrum inhibitor of viral RNA polymerase. Proc. Jpn. Acad. B..

[70] Naesens L., Guddat L.W., Keough D.T., Van Kuilenburg A.B.P., Meijer J., Voorde J.V., Balzarini J. (2013). Role of human hypoxanthine guanine phosphoribosyltransferase in activation of the antiviral agent T-705 (favipiravir). Mol. Pharmacol..

[71] Furuta Y., Takahashi K., Shiraki K., Sakamoto K., Smee D.F., Barnard D.L., Gowen B.B., Julander J.G., Morrey J.D. (2009). T-705 (favipiravir) and related compounds: Novel broad-spectrum inhibitors of RNA viral infections. Antiviral Res..

[72] Sissoko D., Laouenan C., Folkesson E., M’Lebing A.B., Beavogui A.H., Baize S., Camara A.-M., Maes P., Shepherd S., Danel C. (2016). Experimental treatment with favipiravir for Ebola virus disease (the JIKI Trial): A historically controlled, single-arm proof-of-concept trial in Guinea. PLoS Med..

[73] Cai Q., Yang M., Liu D., Chen J., Shu D., Xia J., Liao X., Gu Y., Cai Q., Yang Y. (2020). Experimental Treatment with Favipiravir for COVID-19: An Open-Label Control Study. Engineering.

[74] Chen C., Huang J., Yin P., Zhang Y., Cheng Z.-S., Wu J., Chen S., Zhang Y., Chen B., Lu M. (2020). Favipiravir versus arbidol for COVID-19: A randomized clinical trial. medRxiv.

[75] Naksuk N., Lazar S., Peeraphatdit T.B. (2020). Cardiac safety of off-label COVID-19 drug therapy: A review and proposed monitoring protocol. Eur. Heart J. Acute Cardiovasc. Care.

[76] Prescribing information for Avigan tablet 200 mg. https://www.cdc.gov.tw/File/Get/ht8jUiB_MI-aKnlwstwzvw.

[77] ATEA Pharmaceuticals AT-527. https://ateapharma.com/at-527/.

[78] Good S.S., Moussa A., Zhou X.J., Pietropaolo K., Sommadossi J.P. (2020). Preclinical evaluation of AT-527, a novel guanosine nucleotide prodrug with potent, pan-genotypic activity against hepatitis C virus. PLoS ONE.

[79] Berliba E., Bogus M., Vanhoutte F., Berghmans P.J., Good S.S., Moussa A., Pietropaolo K., Murphy R.L., Zhou X.J., Sommadossi J.P. (2019). Safety, pharmacokinetics and antiviral activity of AT-527, a novel purine nucleotide prodrug, in HCV-infected subjects with and without cirrhosis. Antimicrob. Agents Chemother..

[80] Sheahan T.P., Sims A.C., Zhou S., Graham R.L., Pruijssers A.J., Agostini M.L., Leist S.R., Schäfer A., Dinnon K.H., Stevens L.J. (2020). An orally bioavailable broad-spectrum antiviral inhibits SARS-CoV-2 in human airway epithelial cell cultures and multiple coronaviruses in mice. Sci. Transl. Med..

[81] Toots M., Yoon J.J., Cox R.M., Hart M., Sticher Z.M., Makhsous N., Plesker R., Barrena A.H., Reddy P.G., Mitchell D.G. (2019). Characterization of orally efficacious influenza drug with high resistance barrier in ferrets and human airway epithelia. Sci. Transl. Med..

[82] Hampton T. (2020). New Flu Antiviral Candidate May Thwart Drug Resistance. JAMA.

[83] Lv Z., Chu Y., Wang Y. (2015). HIV protease inhibitors: A review of molecular selectivity and toxicity. HIV/AIDS.

[84] AbbVie Kaletra Prescribing Information 2020. www.rxabbvie.com/pdf/kaletratabpi.pdf.

[85] Choy K.-T., Wong A.Y.-L., Kaewpreedee P., Sia S.-F., Chen D., Hui K.P.Y., Chu D.K.W., Chan M.C.W., Cheung P.P.-H., Huang X. (2020). Remdesivir, lopinavir, emetine, and homoharringtonine inhibit SARS-CoV-2 replication in vitro. Antiviral Res..

[86] Wang Q., Zhao Y., Chen X., Hong A. (2020). Virtual Screening of Approved Clinic Drugs with Main Protease (3CLpro) Reveals Potential Inhibitory Effects on SARS-CoV-2. J. Biomol. Struct. Dyn..

[87] Liu X., Wang X.-J. (2020). Potential inhibitors against 2019-nCoV coronavirus M protease from clinically approved medicines. J. Genet. Genom..

[88] Chu C.M., Cheng V.C., Hung I.F., Wong M.M., Chan K.H., Chan K.S., Kao R.Y., Poon L.L., Wong C.L., Guan Y. (2004). Role of lopinavir/ritonavir in the treatment of SARS: Initial virological and clinical findings. Thorax.

[89] Chen F., Chan K.H., Jiang Y., Kao R.Y., Lu H.T., Fan K.W., Cheng V.C., Tsui W.H., Hung I.F., Lee T.S. (2004). In vitro susceptibility of 10 clinical isolates of SARS coronavirus to selected antiviral compounds. J. Clin. Virol..

[90] Yao T.T., Qian J.D., Zhu W.Y., Wang Y., Wang G.Q. (2020). A systematic review of lopinavir therapy for SARS coronavirus and MERS coronavirus-A possible reference for coronavirus disease-19 treatment option. J. Med. Virol..

[91] Chan J.F., Yao Y., Yeung M.L., Deng W., Bao L., Jia L., Li F., Xiao C., Gao H., Yu P. (2015). Treatment with Lopinavir/Ritonavir or Interferon-β1b Improves Outcome of MERS-CoV Infection in a Nonhuman Primate Model of Common Marmoset. J. Infect. Dis..

[92] Martinez M.A. (2020). Compounds with therapeutic potential against novel respiratory 2019 coronavirus. Antimicrob. Agents Chemother..

[93] Kim U.J., Won E.J., Kee S.J., Jung S.I., Jang H.C. (2016). Combination therapy with lopinavir/ritonavir, ribavirin and interferon-α for Middle East respiratory syndrome. Antivir Ther..

[94] Lim J., Jeon S., Shin H.Y., Kim M.J., Seong Y.M., Lee W.J., Choe K.W., Kang Y.M., Lee B., Park S.J. (2020). Case of the index patient who caused tertiary transmission of coronavirus disease 2019 in Korea: The application of lopinavir/ritonavir for the treatment of COVID-19 pneumonia monitored by quantitative RT-PCR. J. Korean Med. Sci..

[95] Cao B., Wang Y., Wen D., Liu W., Wang J., Fan G., Ruan L., Song B., Cai Y., Wei M. (2020). A Trial of Lopinavir-Ritonavir in Adults Hospitalized with Severe Covid-19. N. Engl. J. Med..

[96] Deng L., Li C., Zeng Q., Liu X., Li X., Zhang H., Hong Z., Xia J. (2020). Arbidol combined with LPV/r versus LPV/r alone against Corona Virus Disease 2019: A retrospective cohort study. J. Infect..

[97] Liu F., Xu A., Zhang Y., Xuan W., Yan T., Pan K., Yu W., Zhang J. (2020). Patients of COVID-19 may benefit from sustained Lopinavir-combined regimen and the increase of Eosinophil may predict the outcome of COVID-19 progression. Int. J. Infect. Dis..

[98] Young B.E., Ong S.W.X., Kalimuddin S., Low J.G., Tan S.Y., Loh J., Ng O.-T., Marimuthu K., Ang L.W., Mak T.M. (2020). Epidemiologic Features and Clinical Course of Patients Infected With SARS-CoV-2 in Singapore. JAMA.

[99] Zhou F., Yu T., Du R., Fan G., Liu Y., Liu Z., Xiang J., Wang Y., Song B., Gu X. (2020). Clinical course and risk factors for mortality of adult inpatients with COVID-19 in Wuhan, China: A retrospective cohort study. Lancet.

[100] Huang M., Tang T., Pang P., Li M., Ma R., Lu J., Shu J., You Y., Chen B., Liang J. (2020). Treating COVID-19 with Chloroquine. J. Mol. Cell Biol..

[101] De Meyer S., Azijn H., Surleraux D., Jochmans D., Tahri A., Pauwels R., Wigerinck P., De Béthune M.P. (2005). TMC114, a novel human immunodeficiency virus type 1 protease inhibitor active against protease inhibitor-resistant viruses, including a broad range of clinical isolates. Antimicrob. Agents Chemother..

[102] Davis D.A., Soule E.E., Davidoff K.S., Daniels S.I., Naiman N.E., Yarchoan R. (2012). Activity of human immunodeficiency virus type 1 protease inhibitors against the initial autocleavage in Gag-Pol polyprotein processing. Antimicrob. Agents Chemother..

[103] Purohit R., Sethumadhavan R. (2009). Structural basis for the resilience of Darunavir (TMC114) resistance major flap mutations of HIV-1 protease. Interdiscip. Sci..

[104] Yamamoto N., Yang R., Yoshinaka Y., Amari S., Nakano T., Cinatl J., Rabenau H., Doerr H.W., Hunsmann G., Otaka A. (2004). HIV protease inhibitor nelfinavir inhibits replication of SARS-associated coronavirus. Biochem. Biophys. Res. Commun..

[105] De Meyer S., Bojkova D., Cinatl J., Van Damme E., Buyck C., Van Loock M., Woodfall B., Ciesek S. (2020). Lack of antiviral activity of darunavir against SARS-CoV-2. Int. J. Infect. Dis..

[106] The National Institutes of Health COVID-19 Treatment Guidelines Panel. Coronavirus Disease 2019 (COVID-19) Treatment Guidelines. https://www.covid19treatmentguidelines.nih.gov/.

[107] Dierynck I., Van Marck H., Van Ginderen M., Jonckers T.H., Nalam M.N., Schiffer C.A., Raoof A., Kraus G., Picchio G. (2011). TMC310911, a novel human immunodeficiency virus type 1 protease inhibitor, shows in vitro an improved resistance profile and higher genetic barrier to resistance compared with current protease inhibitors. Antimicrob. Agents Chemother..

[108] Stellbrink H.J., Arastéh K., Schürmann D., Stephan C., Dierynck I., Smyej I., Hoetelmans R.M., Truyers C., Meyvisch P., Jacquemyn B. (2014). Antiviral activity, pharmacokinetics, and safety of the HIV-1 protease inhibitor TMC310911, coadministered with ritonavir, in treatment-naive HIV-1-infected patients. J. Acquir. Immune Defic. Syndr..

[109] Fintelman-Rodrigues N., Sacramento C.Q., Lima C.R., Da Silva F.S., Ferreira A.C., Mattos M., De Freitas C.S., Soares V.C., Dias S.D.S.G., Temerozo J.R. (2020). Atazanavir, alone or in combination with ritonavir, inhibits SARS-CoV-2 replication and pro-inflammatory cytokine production. Antimicrob. Agents Chemother..

[110] Yamamoto N., Matsuyama S., Hoshino T., Yamamoto N. (2020). Nelfinavir inhibits replication of severe acute respiratory syndrome coronavirus 2 in vitro. bioRxiv.

[111] Borgio J.F., Alsuwat H.S., Al Otaibi W.M., Ibrahim A.M., Almandil N.B., Al Asoom L.I., Salahuddin M., Kamaraj B., AbdulAzeez S. (2020). State-of-the-art tools unveil potent drug targets amongst clinically approved drugs to inhibit helicase in SARS-CoV-2. Arch. Med. Sci..

[112] Jiang Y., Andrews S.W., Condroski K.R., Buckman B., Serebryany V., Wenglowsky S., Kennedy A.L., Madduru M.R., Wang B., Lyon M. (2014). Discovery of danoprevir (ITMN-191/R7227), a highly selective and potent inhibitor of hepatitis C virus (HCV) NS3/4A protease. J. Med. Chem..

[113] Nguyen D.D., Gao K., Chen J., Wang R., Wei G. (2020). Potentially highly potent drugs for 2019-nCoV. bioRxiv.

[114] Brown A.J., Won J.J., Graham R.L., Dinnon K.H., Sims A.C., Feng J.Y., Cihlar T., Denison M.R., Baric R.S., Sheahan T.P. (2019). Broad spectrum antiviral remdesivir inhibits human endemic and zoonotic deltacoronaviruses with a highly divergent RNA dependent RNA polymerase. Antiviral Res..

[115] Chen H., Zhang Z., Wang L., Huang Z., Gong F., Li X., Chen Y., Wu J.J. (2020). First Clinical Study Using HCV Protease Inhibitor Danoprevir to Treat Naive and Experienced COVID-19 Patients. medRxiv.

[116] MacArthur R.D., Novak R.M. (2008). Reviews of anti-infective agents: Maraviroc: The first of a new class of antiretroviral agents. Clin. Infect. Dis..

[117] Shamsi A., Mohammad T., Anwar S., Alajmi M.F., Hussain A., Rehman M.T., Islam A., Hassan M.I. (2020). Glecaprevir and Maraviroc are high-affinity inhibitors of SARS-CoV-2 main protease: Possible implication in COVID-19 therapy. Biosci. Rep..

[118] Adedeji A.O., Severson W., Jonsson C., Singh K., Weiss S.R., Sarafianos S.G. (2013). Novel inhibitors of severe acute respiratory syndrome coronavirus entry that act by three distinct mechanisms. J. Virol..

[119] Syed Y.Y. (2019). Selinexor: First Global Approval. Drugs.

[120] Podar K., Shah J., Chari A., Richardson P.G., Jagannath S. (2020). Selinexor for the treatment of multiple myeloma. Expert Opin. Pharmacother..

[121] Chari A., Vogl D.T., Gavriatopoulou M., Nooka A.K., Yee A.J., Huff C.A., Moreau P., Dingli D., Cole C., Lonial S. (2019). Oral selinexor-dexamethasone for triple-class refractory multiple myeloma. N. Engl. J. Med..

[122] Widman D.G., Gornisiewicz S., Shacham S., Tamir S. (2018). In vitro toxicity and efficacy of verdinexor, an exportin 1 inhibitor, on opportunistic viruses affecting immunocompromised individuals. PLoS ONE.

[123] Mathew C., Ghildyal R. (2017). CRM1 inhibitors for antiviral therapy. Front Microbiol..

[124] Gordon D.E., Jang G.M., Bouhaddou M., Xu J., Obernier K., O’Meara M.J., Guo J.Z., Swaney D.L., Tummino T.A., Hüttenhain R. (2020). A SARS-CoV-2 protein interaction map reveals targets for drug repurposing. Nature.

[125] Freundt E.C., Yu L., Park E., Lenardo M.J., Xu X.N. (2009). Molecular determinants for subcellular localization of the severe acute respiratory syndrome coronavirus open reading frame 3b protein. J. Virol..

[126] Sharma K., Åkerström S., Sharma A.K., Chow V.T., Teow S., Abrenica B., Booth S.A., Booth T.F., Mirazimi A., Lal S.K. (2011). SARS-CoV 9b protein diffuses into nucleus, undergoes active Crm1 mediated nucleocytoplasmic export and triggers apoptosis when retained in the nucleus. PLoS ONE.

[127] McBride R., Van Zyl M., Fielding B.C. (2014). The coronavirus nucleocapsid is a multifunctional protein. Viruses.

[128] Ujike M., Huang C., Shirato K., Matsuyama S., Makino S., Taguchi F. (2012). Two palmitylated cysteine residues of the severe acute respiratory syndrome coronavirus spike (S) protein are critical for S incorporation into virus-like particles, but not for M-S co-localization. J. Gen. Virol..

[129] Sims A.C., Tilton S.C., Menachery V.D., Gralinski L.E., Schäfer A., Matzke M.M., Webb-Robertson B.J., Chang J., Luna M.L., Long C.E. (2013). Release of severe acute respiratory syndrome coronavirus nuclear import block enhances host transcription in human lung cells. J. Virol..

[130] Tajiri N., De La Peña I., Acosta S.A., Kaneko Y., Tamir S., Landesman Y., Carlson R., Shacham S., Borlongan C.V. (2016). A Nuclear Attack on Traumatic Brain Injury: Sequestration of Cell Death in the Nucleus. CNS Neurosci Ther..

[131] Perwitasari O., Johnson S., Yan X., Howerth E., Shacham S., Landesman Y., Baloglu E., McCauley D., Tamir S., Tompkins S.M. (2014). Verdinexor, a novel selective inhibitor of nuclear export, reduces influenza a virus replication in vitro and in vivo. J. Virol..

[132] Wu M., Gui H., Feng Z., Xu H., Li G., Li M., Chen T., Wu Y., Huang J., Bai Z. (2018). KPT-330, a potent and selective CRM1 inhibitor, exhibits anti-inflammation effects and protection against sepsis. Biochem. Biophys. Res. Commun..

[133] Anderson V.R., Curran M.P. (2007). Nitazoxanide: A review of its use in the treatment of gastrointestinal infections. Drugs.

[134] Shakya A., Bhat H.R., Ghosh S.K. (2018). Update on Nitazoxanide: A Multifunctional Chemotherapeutic Agent. Curr. Drug Discov. Technol..

[135] Rossignol J.F. (2014). Nitazoxanide: A first-in-class broad-spectrum antiviral agent. Antiviral Res..

[136] Rossignol J.F. (2016). Nitazoxanide, a new drug candidate for the treatment of Middle East respiratory syndrome coronavirus. J. Infect. Public Health.

[137] Sisson G., Goodwin A., Raudonikiene A., Hughes N.J., Mukhopadhyay A.K., Berg D.E., Hoffman P.S. (2002). Enzymes associated with reductive activation and action of nitazoxanide, nitrofurans, and metronidazole in Helicobacter pylori. Antimicrob. Agents Chemother..

[138] Rossignol J.F., La Frazia S., Chiappa L., Ciucci A., Santoro M.G. (2009). Thiazolides, a new class of anti-influenza molecules targeting viral hemagglutinin at post-translational level. J. Biol. Chem..

[139] Cao J., Forrest J.C., Zhang X. (2015). A screen of the NIH collection small molecule library identifies potential coronavirus. Antiviral Res..

[140] Piacentini S., La Frazia S., Riccio A., Pedersen J.Z., Topai A., Nicolotti O., Rossignol J.-F., Santoro M.G. (2018). Nitazoxanide inhibits paramyxovirus replication by targeting the Fusion protein folding: Role of glycoprotein-specific thiol oxidoreductase ERp57. Sci. Rep..

[141] Miner K., Labitzke K., Liu B., Wang P., Henckels K., Gaida K., Elliott R., Chen J.J., Liu L., Leith A. (2019). Drug repurposing: The anthelmintics niclosamide and nitazoxanide are potent TMEM16A antagonists that fully bronchodilate airways. Front Pharmacol..

[142] Lucas S. (2016). The Pharmacology of Indomethacin. Headache.

[143] Amici C., Di Caro A., Ciucci A., Chiappa L., Castilletti C., Martella V., Decaro N., Buonavoglia C., Capobianchi M.R., Santoro M.G. (2006). Indomethacin has a potent antiviral activity against SARS coronavirus. Antivir. Ther..

[144] Xu T., Gao X., Wu Z., Selinger D.W., Zhou Z. (2020). Indomethacin has a potent antiviral activity against SARS CoV-2 in vitro and canine coronavirus in vivo. bioRxiv.

[145] Amici C., La Frazia S., Brunelli C., Balsamo M., Angelini M., Santoro M.G. (2015). Inhibition of viral protein translation by indomethacin in vesicular stomatitis virus infection: Role of eIF2α kinase PKR. Cell Microbiol..

[146] Rossen J.W., Bouma J., Raatgeep R.H., Büller H.A., Einerhand A.W. (2004). Inhibition of cyclooxygenase activity reduces rotavirus infection at a postbinding step. J. Virol..

[147] Bai J.Y., Liu B.H., Zhao D.Y., Cheng G.F. (2003). Effect of indomethacin on expression of interleukin-6 caused by lipopolysaccharide in rheumatoid arthritic patients’ synoviocyte. Acta Pharm. Sin..

[148] Todd P.A., Clissold S.P. (1990). Naproxen. A reappraisal of its pharmacology, and therapeutic use in rheumatic diseases and pain states. Drugs.

[149] Kearney P.M., Baigent C., Godwin J., Halls H., Emberson J.R., Patrono C. (2006). Do selective cyclo-oxygenase-2 inhibitors and traditional non-steroidal anti-inflammatory drugs increase the risk of atherothrombosis? Meta-analysis of randomised trials. BMJ.

[150] Zheng W., Fan W., Zhang S., Jiao P., Shang Y., Cui L., Mahesutihan M., Li J., Wang D., Gao G.F. (2019). Naproxen Exhibits Broad Anti-influenza Virus Activity in Mice by Impeding Viral Nucleoprotein Nuclear Export. Cell Rep..

[151] Ebell M.H. (2017). In Hospitalized Patients with Influenza and an Infiltrate, Adding Clarithromycin and Naproxen to Oseltamivir Improves Outcomes. Am. Fam. Physician.

[152] Pan T., Peng Z., Tan L., Zou F., Zhou N., Liu B., Liang L., Chen C., Liu J., Wu L. (2018). Nonsteroidal Anti-inflammatory Drugs Potently Inhibit the Replication of Zika Viruses by Inducing the Degradation of AXL. J. Virol..

[153] Terrier O., Dilly S., Pizzorno A., Henri J., Berenbaum F., Lina B., Fève B., Adnet F., Sabbah M., Rosa-Calatrava M. (2020). Broad-spectrum antiviral activity of naproxen: From Influenza A to SARS-CoV-2 Coronavirus. bioRxiv.

[154] Immunic Therapeutics IMU-838. https://www.immunic-therapeutics.com/imu838/.

[155] Muehler A., Peelen E., Kohlhof H., Gröppel M., Vitt D. (2020). Vidofludimus calcium, a next generation DHODH inhibitor for the Treatment of relapsing-remitting multiple sclerosis. Mult. Scler. Relat. Disord..

[156] Muehler A., Kohlhof H., Groeppel M., Vitt D. (2020). Safety, Tolerability and Pharmacokinetics of Vidofludimus calcium (IMU-838) After Single and Multiple Ascending Oral Doses in Healthy Male Subjects. Eur. J. Drug Metab. Pharmacokinet..

[157] Immunic Therapeutics. Immunic, Inc. Reports that IMU-838, a Selective Oral DHODH Inhibitor, Has Demonstrated Preclinical Activity Against SARS-CoV-2 and Explores Plans for a Phase 2 Clinical Trial in COVID-19 Patients. https://www.immunic-therapeutics.com/2020/04/21/immunic-inc-reports-that-imu-838-a-selective-oral-dhodh-inhibitor-has-demonstrated-preclinical-activity-against-sars-cov-2-and-explores-plans-for-a-phase-2-clinical-trial-in-covid-19-patients/.

[158] Peters G.J. (2018). Re-evaluation of Brequinar sodium, a dihydroorotate dehydrogenase inhibitor. Nucleosides Nucleotides Nucleic Acids.

[159] Vyas V.K., Ghate M. (2011). Recent developments in the medicinal chemistry and therapeutic potential of dihydroorotate dehydrogenase (DHODH) inhibitors. Mini-Rev. Med. Chem..

[160] Madak J.T., Bankhead A., Cuthbertson C.R., Showalter H.D., Neamati N. (2019). Revisiting the role of dihydroorotate dehydrogenase as a therapeutic target for cancer. Pharmacol. Ther..

[161] Boschi D., Pippione A.C., Sainas S., Lolli M.L. (2019). Dihydroorotate dehydrogenase inhibitors in anti-infective drug research. Eur. J. Med. Chem..

[162] Park J.G., Ávila-Pérez G., Nogales A., Blanco-Lobo P., De La Torre J.C., Martínez-Sobrido L. (2020). Identification and Characterization of Novel Compounds with Broad-Spectrum Antiviral Activity against Influenza A and B Viruses. J. Virol..

[163] Andersen P.I., Krpina K., Ianevski A., Shtaida N., Jo E., Yang J., Koit S., Tenson T., Hukkanen V., Anthonsen M.W. (2019). Novel Antiviral Activities of Obatoclax, Emetine, Niclosamide, Brequinar, and Homoharringtonine. Viruses.

[164] Li S.F., Gong M.J., Sun Y.F., Shao J.J., Zhang Y.G., Chang H.Y. (2019). Antiviral activity of brequinar against foot-and-mouth disease virus infection in vitro and in vivo. Biomed. Pharmacother..

[165] Xiong R., Zhang L., Li S., Sun Y., Ding M., Wang Y., Zhao Y., Wu Y., Shang W., Jiang X. (2020). Novel and potent inhibitors targeting DHODH, a rate-limiting enzyme in de novo pyrimidine biosynthesis, are broad-spectrum antiviral against RNA viruses including newly emerged coronavirus SARS-CoV-2. bioRxiv.

[166] Langtry H.D., Grant S.M., Goa K.L. (1989). Famotidine. An updated review of its pharmacodynamic and pharmacokinetic properties, and therapeutic use in peptic ulcer disease and other allied diseases. Drugs.

[167] Freedberg D.E., Conigliaro J., Wang T.C., Tracey K.J., Callahan M.V., Abrams J.A., Sobieszczyk M.E., Markowitz D.D., Gupta A., O’Donnell M.R. (2020). Famotidine Use is Associated with Improved Clinical Outcomes in Hospitalized COVID-19 Patients: A Propensity Score Matched Retrospective Cohort Study. Gastroenterology.

[168] Janowitz T., Gablenz E., Pattinson D., Wang T.C., Conigliaro J., Tracey K., Tuveson D. (2020). Famotidine use and quantitative symptom tracking for COVID-19 in non-hospitalised patients: A case series. Gut.

[169] Wu C., Liu Y., Yang Y., Zhang P., Zhong W., Wang Y., Wang Q., Xu Y., Li M., Li X. (2020). Analysis of therapeutic targets for SARS-CoV-2 and discovery of potential drugs by computational methods. Acta Pharm. Sin. B.

[170] Wang Q., Arnst K.E., Wang Y., Kumar G., Ma D., Chen H., Wu Z., Yang J., White S.W., Miller D.D. (2018). Structural modification of the 3,4,5-trimethoxyphenyl moiety in the tubulin inhibitor VERU-111 leads to improved antiproliferative activities. J. Med. Chem..

[171] Veru Inc. VERU-111. https://verupharma.com/pipeline/veru-111/.

[172] Deftereos S.G., Giannopoulos G., Vrachatis D.A., Siasos G.D., Giotaki S.G., Gargalianos P., Metallidis S., Sianos G., Baltagiannis S., Panagopoulos P. (2020). Effect of Colchicine vs Standard Care on Cardiac and Inflammatory Biomarkers and Clinical Outcomes in Patients Hospitalized With Coronavirus Disease 2019: The GRECCO-19 Randomized Clinical Trial. JAMA.

[173] Li E.K., Tam L.S., Tomlinson B. (2004). Leflunomide in the treatment of rheumatoid arthritis. Clin. Ther..

[174] Breedveld F.C., Dayer J.M. (2000). Leflunomide: Mode of action in the treatment of rheumatoid arthritis. Ann. Rheum. Dis..

[175] Herrmann M.L., Schleyerbach R., Kirschbaum B.J. (2000). Leflunomide: An immunomodulatory drug for the treatment of rheumatoid arthritis and other autoimmune diseases. Immunopharmacology.

[176] Bernhoff E., Tylden G.D., Kjerpeseth L.J., Gutteberg T.J., Hirsch H.H., Rinaldo C.H. (2010). Leflunomide inhibition of BK virus replication in renal tubular epithelial cells. J. Virol..

[177] Avery R.K., Mossad S.B., Poggio E., Lard M., Budev M., Bolwell B., Waldman W.J., Braun W., Mawhorter S.D., Fatica R. (2010). Utility of leflunomide in the treatment of complex cytomegalovirus syndromes. Transplantation.

[178] Teschner S., Burst V. (2010). Leflunomide: A drug with a potential beyond rheumatology. Immunotherapy.

[179] Fox R.I., Herrmann M.L., Frangou C.G., Wahl G.M., Morris R.E., Strand V., Kirschbaum B.J. (1999). Mechanism of action for leflunomide in rheumatoid arthritis. Clin. Immunol..

[180] Sehgal S.N. (2003). Sirolimus: Its discovery, biological properties, and mechanism of action. Transplant Proc..

[181] Kirken R.A., Wang Y.L. (2003). Molecular actions of sirolimus: Sirolimus and mTor. Transplant Proc..

[182] Stohr S., Costa R., Sandmann L., Westhaus S., Pfaender S., Anggakusuma, Dazert E., Meuleman P., Vondran F.W.R., Manns M.P. (2016). Host cell mTORC1 is required for HCV RNA replication. Gut.

[183] Kindrachuk J., Ork B., Hart B.J., Mazur S., Holbrook M.R., Frieman M.B., Traynor D., Johnson R.F., Dyall J., Kuhn J.H. (2015). Antiviral potential of ERK/MAPK and PI3K/AKT/mTOR signaling modulation for middle east respiratory syndrome coronavirus infection as identified by tem-poral kinome analysis. Antimicrob. Agents Chemother..

[184] Zhou Y., Hou Y., Shen J., Huang Y., Martin W., Cheng F. (2020). Network-based drug repurposing for novel coronavirus 2019-nCoV/SARS-CoV-2. Cell Discov..

[185] Wang C.H., Chung F.T., Lin S.M., Huang S.Y., Chou C.L., Lee K.Y., Lin T.Y., Kuo H.P. (2014). Adjuvant treatment with a mammalian target of rapamycin inhibitor, sirolimus, and steroids improves outcomes in patients with severe H1N1 pneumonia and acute respiratory failure. Crit. Care Med..

[186] Cragg G.M., Newman D.J. (2004). Marine natural products and related compounds in clinical and advanced preclinical trials. J. Nat. Prod..

[187] Gomes N.G.M., Valentão P., Andrade P.B., Pereira R.B. (2020). Plitidepsin to treat multiple myeloma. Drugs Today (Barc.).

[188] PharmMar PharmaMar Reports Positive Results for Aplidin^®^ against Coronavirus HCoV-229E. http://pharmamar.com/wp-content/uploads/2020/03/PR_Results_Aplidin_coronavirus.pdf.

[189] Sasikumar A.N., Perez W.B., Kinzy T.G. (2012). The many roles of the eukaryotic elongation factor 1 complex. Wiley Interdiscip. Rev..

[190] Li D., Wei T., Abbott C.M., Harrich D. (2013). The unexpected roles of eukaryotic translation elongation factors in RNA virus replication and pathogenesis. Microbiol. Mol. Biol. Rev..

[191] Forsythe P., Paterson S. (2014). Ciclosporin 10 years on: Indications and efficacy. Vet. Rec..

[192] Kapturczak M.H., Meier-Kriesche H.U., Kaplan B. (2004). Pharmacology of calcineurin antagonists. Transplant Proc..

[193] Russell G., Graveley R., Seid J., Al-Humidan A.K., Skjodt H. (1992). Mechanisms of action of cyclosporine and effects on connective tissues. Semin. Arthritis Rheum..

[194] Faulds D., Goa K.L., Benfield P. (1993). Cyclosporin. A review of its pharmacodynamic and pharmacokinetic properties, and therapeutic use in immunoregulatory disorders. Drugs.

[195] The U.S. Food and Drug Administration Prescribing Information for Neoral^®^. https://www.accessdata.fda.gov/drugsatfda_docs/label/2009/050715s027,050716s028lbl.pdf.

[196] De Wilde A.H., Zevenhoven-Dobbe J.C., Van der Meer Y., Thiel V., Narayanan K., Makino S., Snijder E.J., Van Hemert M.J. (2011). Cyclosporin A inhibits the replication of diverse coronaviruses. J. Gen. Virol..

[197] García-Serradilla M., Risco C., Pacheco B. (2019). Drug repurposing for new, efficient, broad spectrum antivirals. Virus Res..

[198] Ianevski A., Zusinaite E., Kuivanen S., Strand M., Lysvand H., Teppor M., Kakkola L., Paavilainen H., Laajala M., Kallio-Kokko H. (2018). Novel activities of safe-in-human broad-spectrum antiviral agents. Antiviral Res..

[199] Cour M., Ovize M., Argaud L. (2020). Cyclosporine A: A valid candidate to treat COVID-19 patients with acute respiratory failure?. Crit. Care.

[200] Di Lernia V. (2020). Antipsoriatic treatments during COVID-19 outbreak. Dermatol. Ther..

[201] Mobarra N., Shanaki M., Ehteram H., Nasiri H., Sahmani M., Saeidi M., Goudarzi M., Pourkarim H., Azad M. (2016). A Review on Iron Chelators in Treatment of Iron Overload Syndromes. Int. J. Hematol. Oncol. Stem Cell Res..

[202] Allain P., Mauras Y., Chaleil D., Simon P., Ang K.S., Cam G., Le Mignon L., Simon M. (1987). Pharmacokinetics and renal elimination of desferrioxamine and ferrioxamine in healthy subjects and patients with haemochromatosis. Br. J. Clin. Pharmacol..

[203] Bataille S., Pedinielli N., Bergounioux J.P. (2020). Could ferritin help the screening for COVID-19 in hemodialysis patients?. Kidney Int..

[204] Drakesmith H., Prentice A. (2008). Viral infection and iron metabolism. Nat. Rev. Microbiol..

[205] Moalem S., Weinberg E.D., Percy M.E. (2004). Hemochromatosis and the enigma of misplaced iron: Implications for infectious disease and survival. Biometals.

[206] Wessling-Resnick M. (2010). Iron homeostasis and the inflammatory response. Annu. Rev. Nutr..

[207] Ali K., Kim R.Y., Brown A.C., Donovan C., Vanka K.S., Mayall J.R., Liu G., Pillar A.L., Jones-Freeman B., Xenaki D. (2020). Critical role for iron accumulation in the pathogenesis of fibrotic lung disease. J. Pathol..

[208] Dalamaga M., Karampela I., Mantzoros C.S. (2020). Commentary: Could Iron Chelators Prove to Be Useful as an Adjunct to COVID-19 Treatment Regimens?. Metabolism.

[209] Williams A., Meyer D. (2009). Desferrioxamine as immunomodulatory agent during microorganism infection. Curr. Pharm. Des..

[210] Georgiou N.A., Van Der Bruggen T., Oudshoorn M., Nottet H.S., Marx J.J., Van Asbeck B.S. (2000). Inhibition of human immunodeficiency virus type 1 replication in human mononuclear blood cells by the iron chelators deferoxamine, deferiprone, and bleomycin. J. Infect. Dis..

[211] Bartolomei G., Cevik R.E., Marcello A. (2011). Modulation of hepatitis C virus replication by iron and hepcidin in Huh7 hepatocytes. J. Gen. Virol..

[212] Theurl I., Zoller H., Obrist P., Datz C., Bachmann F., Elliott R.M., Weiss G. (2004). Iron regulates hepatitis C virus translation via stimulation of expression of translation initiation factor 3. J. Infect. Dis..

[213] Duchemin J., Paradkar P.N. (2017). Iron availability affects West Nile virus infection in its mosquito vector. J. Virol..

[214] Visseren F., Verkerk M.S., Van Der Bruggen T., Marx J.J., Van Asbeck B.S., Diepersloot R.J. (2002). Iron chelation and hydroxyl radical scavenging reduce the inflammatory response of endothelial cells after infection with Chlamydia pneumoniae or influenza A. Eur. J. Clin. Investig..

[215] Artymowicz R.J., James V.E. (1993). Atovaquone: A new antipneumocystis agent. Clin. Pharm..

[216] Haile L.G., Flaherty J.F. (1993). Atovaquone: A review. Ann. Pharmacother..

[217] Behbahani R., Moshfeghi M., Baxter J.D. (1995). Therapeutic approaches for AIDS-related Toxoplasmosis. Ann. Pharmacother..

[218] Raju M., Salazar J.C., Leopold H., Krause P.J. (2000). Atovaquone and azithromycin for the treatment of babesiosis. N. Engl. J. Med..

[219] Spencer C.M., Goa K.L. (1995). Atovaquone. A review of its pharmacological properties and therapeutic efficacy in opportunistic infections. Drugs.

[220] Baggish A.L., Hill D.R. (2002). Antiparasitic agent atovaquone. Antimicrob. Agents Chemother..

[221] Farag A., Wang P., Ahmed M., Sadek H. (2020). Identification of FDA approved drugs targeting COVID-19 virus by structure-based drug repositioning. ChemRxiv.

[222] Popovic M., Stefanovic D., Pejnovic N. (1998). Comparative study of the clinical efficacy of four DMARDs (leflunomide, methotrexate, cyclosporine, and levamisole) in patients with rheumatoid arthritis. Transplant Proc..

[223] Sany J. (1990). Immunological treatment of rheumatoid arthritis. Clin. Exp. Rheumatol..

[224] Olsen N., Halberg P., Halskov O., Bentzon M.W. (1988). Scintimetric assessment of synovitis activity during treatment with disease modifying antirheumatic drugs. Ann. Rheumatol. Dis..

[225] Buchanan J.A., Lavonas E.J. (2012). Agranulocytosis and other consequences due to use of illicit cocaine contaminated with levamisole. Curr. Opin. Hematol..

[226] Martin R.J., Verma S., Levandoski M. (2005). Drug resistance and neurotransmitter receptors of nematodes: Recent studies on the mode of action of levamisole. Parasitology.

[227] Boyer O., Moulder J.K., Grandin L., Somers M.J. (2008). Short- and long-term efficacy of levamisole as adjunctive therapy in childhood nephrotic syndrome. Pediatr. Nephrol..

[228] Arya R., Amit D., Prashar V., Mukesh K. (2020). Potential inhibitors against papain-like protease of novel coronavirus (SARS-CoV-2) from FDA approved drugs. ChemRxiv.

[229] Lee K.C., Ladizinski B., Federman D.G. (2012). Complications associated with use of levamisole-contaminated cocaine: An emerging public health challenge. Mayo. Clin. Proc..

[230] Renoux G. (1980). The general immunopharmacology of levamisole. Drugs.

[231] Ogunbiyi P.O., Conlon P.D., Black W.D., Eyre P. (1988). Levamisole-induced attenuation of alveolar macrophage dysfunction in respiratory virus-infected calves. Int. J. Immunopharmacol..

[232] Barnard D.L., Hubbard V.D., Burton J., Smee D.F., Morrey J.D., Otto M.J., Sidwell R.W. (2004). Inhibition of severe acute respiratory syndrome-associated coronavirus (SARSCoV) by calpain inhibitors and beta-D-N4-hydroxycytidine. Antivir. Chem. Chemother..

[233] Schneider M., Ackermann K., Stuart M., Wex C., Protzer U., Schätzl H.M., Gilch S. (2012). Severe Acute Respiratory Syndrome Coronavirus Replication Is Severely Impaired by MG132 due to Proteasome-Independent Inhibition of M-Calpain. J. Virol..

[234] Blade Therapeutics. https://www.blademed.com/science/.

[235] Heard K. (2008). Acetylcysteine for Acetaminophen Poisoning. N. Engl. J. Med..

[236] Geiler J., Michaelis M., Naczk P., Leutz A., Langer K., Doerr H.-W., Cinatl J. (2010). N-acetyl-l-cysteine (NAC) inhibits virus replication and expression of pro-inflammatory molecules in A549 cells infected with highly pathogenic H5N1 influenza A virus. Biochem. Pharmacol..

[237] Ghezzi P., Ungheri D. (2004). Synergistic Combination of N-Acetylcysteine and Ribavirin to Protect from Lethal Influenza Viral Infection in a Mouse Model. Int. J. Immunopathol. Pharmacol..

[238] De Flora S., Grassi C., Carati L. (1997). Attenuation of influenza-like symptomatology and improvement of cell-mediated immunity with long-term N-acetylcysteine treatment. Eur. Respir. J..

[239] Breitkreutz R., Pittack N., Nebe C.T., Schuster D., Brust J., Beichert M., Hack V., Daniel V., Edler L., Droge W. (2000). Improvement of immune functions in HIV infection by sulfur supplementation: Two randomized trials. J. Mol. Med..

[240] Sadowska A., Verbraecken J., Darquennes K., De Backer W.A. (2006). Role of N-acetylcysteine in the management of COPD. Int. J. Chronic Obstr. Pulm. Dis..

[241] Sadowska A., Manuel-Y-Keenoy B., De Backer W. (2007). Antioxidant and anti-inflammatory efficacy of NAC in the treatment of COPD: Discordant in vitro and in vivo dose-effects: A review. Pulm. Pharmacol. Ther..

[242] Medici T.C., Radielovic P. (1979). Effects of Drugs on Mucus Glycoproteins and Water in Bronchial Secretion. J. Int. Med. Res..

[243] eskey G., Abrahem R., Cao R., Gyurjian K., Islamoglu H., Lucero M., Martinez A., Paredes E., Salaiz O., Robinson B. (2018). Glutathione as a Marker for Human Disease. Adv. Appl. Microbiol..

[244] Park J.H., Kang S.-S., Kim J.Y., Tchah H. (2015). The Antioxidant N-Acetylcysteine Inhibits Inflammatory and Apoptotic Processes in Human Conjunctival Epithelial Cells in a High-Glucose Environment. Investig. Opthalmol. Vis. Sci..

[245] Zhang Q., Ju Y., Ma Y., Wang T. (2018). N-acetylcysteine improves oxidative stress and inflammatory response in patients with community acquired pneumonia. Medicine.

[246] Li S.-Y., Chen C., Zhang H.-Q., Guo H.-Y., Wang H., Wang L., Zhang X., Hua S.-N., Yu J., Xiao P.-G. (2005). Identification of natural compounds with antiviral activities against SARS-associated coronavirus. Antivir. Res..

[247] Bae J.-Y., Lee G.E., Park H., Cho J., Kim Y.-E., Lee J.-Y., Ju C., Kim W.-K., Kim J.I., Park M.-S. (2020). Pyronaridine and artesunate are potential antiviral drugs against COVID-19 and influenza. bioRxiv.

[248] Gendrot M., Duflot I., Boxberger M., Delandre O., Jardot P., Le Bideau M., Andreani J., Fonta I., Mosnier J., Rolland C. (2020). Antimalarial artemisinin-based combination therapies (ACT) and COVID-19 in Africa: In vitro inhibition of SARS-CoV-2 replication by mefloquine-artesunate. Int. J. Infect. Dis..

[249] Cao R., Hu H., Li Y., Wang X., Xu M., Liu J., Zhang H., Yan Y., Zhao L., Li W. (2020). Anti-SARS-CoV-2 Potential of Artemisinins In Vitro. ACS Infect. Dis..

[250] Gluck U., Martin U., Bosse B., Reimer K., Mueller S. (2006). A Clinical Study on the Tolerability of a Liposomal Povidone-Iodine Nasal Spray: Implications for Further Development. ORL.

[251] ggers M., Koburger-Janssen T., Eickmann M., Zorn J. (2018). In Vitro Bactericidal and Virucidal Efficacy of Povidone-Iodine Gargle/Mouthwash against Respiratory and Oral Tract Pathogens. Infect. Dis. Ther..

[252] The FDA Approved Drug Products: Peridex (Chlorhexidine Gluconate) Oral Rinse. https://www.accessdata.fda.gov/drugsatfda_docs/label/2013/019028s020lbl.pdf.

[253] Lim K.S., Kam P.C. (2008). Chlorhexidine-pharmacology and clinical applications. Anaesth Intensive Care.

[254] Mohammadi Z., Abbott P.V. (2009). The properties and applications of chlorhexidine in endodontics. Int. Endod. J..

[255] Karpiński T.M., Szkaradkiewicz A.K. (2015). Chlorhexidine-pharmaco-biological activity and application. Eur. Rev. Med. Pharmacol. Sci..

[256] Oz M., Lorke D.E., Hasan M., Petroianu G.A. (2011). ChemInform Abstract: Cellular and Molecular Actions of Methylene Blue in the Nervous System. Chemin.

[257] Chang L., Yan Y., Wang L. (2020). Coronavirus Disease 2019: Coronaviruses and Blood Safety. Transfus. Med. Rev..

[258] Floyd R.A., Schneider J., Dittmer D.P. (2004). Methylene blue photoinactivation of RNA viruses. Antivir. Res..

[259] Jin C., Yu B., Zhang J., Wu H., Zhou X., Yao H., Liu F., Lu X., Cheng L., Jiang M. (2020). Methylene blue photochemical treatment as a reliable SARS-CoV-2 plasma virus inactivation method for blood safety and convalescent plasma therapy for the COVID-19 outbreak. Res. Square.

[260] Eickmann M., Gravemann U., Handke W., Tolksdorf F., Reichenberg S., Müller T.H., Seltsam A. (2020). Inactivation of three emerging viruses—Severe acute respiratory syndrome corona- virus, Crimean-Congo haemorrhagic fever virus and Nipah virus—In platelet concentrates by ultraviolet C light and in plasma by methylene blue plus visible light. Vox Sang.

[261] Eickmann M., Gravemann U., Handke W., Tolksdorf F., Reichenberg S., Müller T.H., Seltsam A. (2018). Inactivation of Ebola virus and Middle East respiratory syndrome coronavirus in platelet concentrates and plasma by ultraviolet C light and methylene blue plus visible light, respectively. Transfusion.

[262] Papin J.F., Floyd R.A., Dittmer D.P. (2005). Methylene blue photoinactivation abolishes West Nile virus infectivity in vivo. Antivir. Res..

[263] Ginimuge P.R., Jyothi S.D. (2010). Methylene blue: Revisited. J. Anaesthesiol. Clin. Pharmacol..

[264] Preiser J.-C., Lejeune P., Roman A., Carlier E., De Backer D., Leeman M., Kahn R., Vincent J.-L. (1995). Methylene blue administration in septic shock: A clinical trial. Crit. Care Med..

[265] Kwok E.S.H., Howes D. (2006). Use of Methylene Blue in Sepsis: A Systematic Review. J. Intensiv. Care Med..

[266] Lin Z.-H., Wang S.-Y., Chen L.-L., Zhuang J.-Y., Ke Q.-F., Xiao D.-R., Lin W.-P. (2017). Methylene Blue Mitigates Acute Neuroinflammation after Spinal Cord Injury through Inhibiting NLRP3 Inflammasome Activation in Microglia. Front. Cell. Neurosci..

[267] Adler H., Beland J.L., Del-Pan N.C., Kobzik L., Brewer J.P., Martin T.R., Rimm I.J. (1997). Suppression of Herpes Simplex Virus Type 1 (HSV-1)–induced Pneumonia in Mice by Inhibition of Inducible Nitric Oxide Synthase (iNOS, NOS2). J. Exp. Med..

[268] Lane T.E., Paoletti A.D., Buchmeier M.J. (1997). Disassociation between the in vitro and in vivo effects of nitric oxide on a neurotropic murine coronavirus. J. Virol..

[269] Pope M., Marsden P.A., Cole E., Sloan S., Fung L.S., Ning Q., Ding J.W., Leibowitz J.L., Phillips M.J., Levy G.A. (1998). Resistance to Murine Hepatitis Virus Strain 3 Is Dependent on Production of Nitric Oxide. J. Virol..

[270] Åkerström S., Mousavi-Jazi M., Klingström J., Leijon M., Lundkvist A., Mirazimi A. (2005). Nitric Oxide Inhibits the Replication Cycle of Severe Acute Respiratory Syndrome Coronavirus. J. Virol..

[271] Åkerström S., Gunalan V., Keng C.T., Tan Y.-J., Mirazimi A. (2009). Dual effect of nitric oxide on SARS-CoV replication: Viral RNA production and palmitoylation of the S protein are affected. Virology.

[272] Colasanti M., Persichini T., Venturini G., Ascenzi P. (1999). S-nitrosylation of viral proteins: Molecular bases for antiviral effect of nitric oxide. IUBMB Life.

[273] Kobayashi J., Murata I. (2020). Nitric oxide inhalation as an interventional rescue therapy for COVID-19-induced acute respiratory distress syndrome. Ann. Intensiv. Care.

[274] Chen L., Liu P., Gao H., Sun B., Chao D., Wang F., Zhu Y., Hedenstierna G., Wang C.G. (2004). Inhalation of Nitric Oxide in the Treatment of Severe Acute Respiratory Syndrome: A Rescue Trial in Beijing. Clin. Infect. Dis..

[275] Zamanian R.T., Pollack C.V., Gentile M.A., Rashid M., Fox J.C., Mahaffey K.W., de Jesus Perez V. (2020). Outpatient Inhaled Nitric Oxide in a Patient with Vasoreactive Idiopathic Pulmonary Arterial Hypertension and COVID-19 Infection. Am. J. Respir. Crit. Care Med..

[276] Marinella M.A. (2020). Indomethacin and resveratrol as potential treatment adjuncts for SARS-CoV-2/COVID-19. Int. J. Clin. Pract..

[277] The National Library of Medicine Pubchem. https://pubchem.ncbi.nlm.nih.gov/compound/445154.

[278] Campagna M., Rivas C. (2010). Antiviral activity of resveratrol. Biochem. Soc. Trans..

[279] Lin S.-C., Ho C.-T., Chuo W.-H., Li S., Wang T.T., Lin C.-C. (2017). Effective inhibition of MERS-CoV infection by resveratrol. BMC Infect. Dis..

[280] Zhao X., Xu J., Song X., Jia R., Yin Z.-Q., Cheng A., Jia R., Zou Y., Li L., Yin L. (2016). Antiviral effect of resveratrol in ducklings infected with virulent duck enteritis virus. Antivir. Res..

[281] Zhao X., Cui Q., Fu Q., Song X., Jia R., Yang Y., Zou Y., Li L., He C., Liang X. (2017). Antiviral properties of resveratrol against pseudorabies virus are associated with the inhibition of IkB kinase activation. Sci. Rep..

[282] Debiaggi M., Tateo F., Pagani L., Luini M., Romero E. (1990). Effects of propolis flavonoids on virus infectivity and replication. Microbiologica.

[283] De Palma A.M., Vliegen I., De Clercq E., Neyts J. (2008). Selective inhibitors of picornavirus replication. Med. Res. Rev..

[284] Ishitsuka H., Ohsawa C., Ohiwa T., Umeda I., Suhara Y. (1982). Antipicornavirus flavone Ro 09-0179. Antimicrob. Agents Chemother..

[285] Kaul T.N., Middleton E., Ogra P.L. (1985). Antiviral effect of flavonoids on human viruses. J. Med. Virol..

[286] Evers D.L., Chao C.-F., Wang X., Zhang Z., Huong S.-M., Huang E.-S. (2005). Human cytomegalovirus-inhibitory flavonoids: Studies on antiviral activity and mechanism of action. Antivir. Res..

[287] Zandi K., Teoh B.-T., Sam S.-S., Wong P.F., Mustafa M.R., Abubakar S. (2011). Antiviral activity of four types of bioflavonoid against dengue virus type-2. Virol. J..

[288] Biancatelli R.M.L.C., Berrill M., Catravas J.D., Marik P.E. (2020). Quercetin and Vitamin C: An Experimental, Synergistic Therapy for the Prevention and Treatment of SARS-CoV-2 Related Disease (COVID-19). Front. Immunol..

[289] Jo S., Kim S., Kim D.Y., Kim M.-S., Shin D.H. (2020). Flavonoids with inhibitory activity against SARS-CoV-2 3CLpro. J. Enzym. Inhib. Med. Chem..

[290] Garaci E. (2007). Thymosin 1: A Historical Overview. Ann. N. Y. Acad. Sci..

[291] Camerini R., Garaci E. (2015). Historical review of thymosin ? 1 in infectious diseases. Expert Opin. Boil. Ther..

[292] Gramenzi A., Cursaro C., Andreone P., Bernardi M. (1998). Thymalfasin: Clinical pharmacology and antiviral applications. BioDrugs.

[293] SciClone Pharmaceuticals. http://www.shijiebiaopin.net/upload/product/2011121219115812.PDF.

[294] Actor J.K., Hwang S.-A., Kruzel M.L. (2009). Lactoferrin as a natural immune modulator. Curr. Pharm. Des..

[295] Cutone A., Rosa L., Ianiro G., Lepanto M.S., Di Patti M.C.B., Valenti P., Musci G. (2020). Lactoferrin’s Anti-Cancer Properties: Safety, Selectivity, and Wide Range of Action. Biomolecules.

[296] Cutone A., Lepanto M.S., Rosa L., Scotti M.J., Rossi A., Ranucci S., De Fino I., Bragonzi A., Valenti P., Musci G. (2019). Aerosolized Bovine Lactoferrin Counteracts Infection, Inflammation and Iron Dysbalance in A Cystic Fibrosis Mouse Model of Pseudomonas aeruginosa Chronic Lung Infection. Int. J. Mol. Sci..

[297] Manzoni P., Meyer M., Stolfi I., Rinaldi M., Cattani S., Pugni L., Romeo M.G., Messner H., Decembrino L., Laforgia N. (2014). Bovine lactoferrin supplementation for prevention of necrotizing enterocolitis in very-low-birth-weight neonates: A randomized clinical trial. Early Hum. Dev..

[298] Berlutti F., Pantanella F., Natalizi T., Frioni A., Paesano R., Polimeni A., Valenti P. (2011). Antiviral Properties of Lactoferrin—A Natural Immunity Molecule. Molecules.

[299] Van Der Strate B., Beljaars L., Molema G., Harmsen M., Meijer D. (2001). Antiviral activities of lactoferrin. Antivir. Res..

[300] Lang J., Yang N., Deng J., Liu K., Yang P., Zhang G., Jiang C. (2011). Inhibition of SARS Pseudovirus Cell Entry by Lactoferrin Binding to Heparan Sulfate Proteoglycans. PLoS ONE.

[301] Peroni D.G., Fanos V. (2020). Lactoferrin is an important factor when breastfeeding and COVID-19 are considered. Acta Paediatr..

[302] Ye M., Fu D., Ren Y., Wang F., Wang D., Zhang F., Xia X., Lv T. (2020). Treatment with convalescent plasma for COVID-19 patients in Wuhan, China. J. Med. Virol..

[303] Sallard E., Lescure F.-X., Yazdanpanah Y., Mentre F., Peiffer-Smadja N., Ader F., Bouadma L., Poissy J., Timsit J.-F., Lina B. (2020). Type 1 interferons as a potential treatment against COVID-19. Antivir. Res..

[304] Davoudi-Monfared E., Rahmani H., Khalili H., Hajiabdolbaghi M., Salehi M., Abbasian L., Kazemzadeh H., Yekaninejad M.S. (2020). A Randomized Clinical Trial of the Efficacy and Safety of Interferon β-1a in Treatment of Severe COVID-19. Antimicrob. Agents Chemother..

[305] Saldanha-Araujo F., Garcez E.M., Silva-Carvalho A.E., Carvalho J.L. (2020). Mesenchymal Stem Cells: A New Piece in the Puzzle of COVID-19 Treatment. Front. Immunol..

