# Supplementary material for: Potential Anti-SARS-CoV-2 Therapeutics That Target the Post-Entry Stages of the Viral Life Cycle: A Comprehensive Review
Source: Viruses. 2020 Sep 26;12(10):1092. doi: 10.3390/v12101092 (PMC7600245; doi:10.3390/v12101092)
Supplement: Supplementary file 1 [file viruses-12-01092-s001.pdf]

Potential Anti-SARS-CoV-2 Therapeutics that Target the Post-Entry Stages of the Viral Life Cycle: A Comprehensive Review

Rami A. Al-Horani[1*] and Srabani Kar[1]

[1]*Division of Basic Pharmaceutical Sciences, College of Pharmacy, Xavier University of Louisiana, New Orleans LA 70125, ralhoran@xula.edu*

* Address for correspondence: Dr. Rami A. Al-Horani, 1 Drexel Drive, College of Pharmacy, New Orleans, LA 70125-1089. Phone: (504) 520-7603, Fax: (504) 520-7954, Email: ralhoran@xula.edu

Supplementary Figures

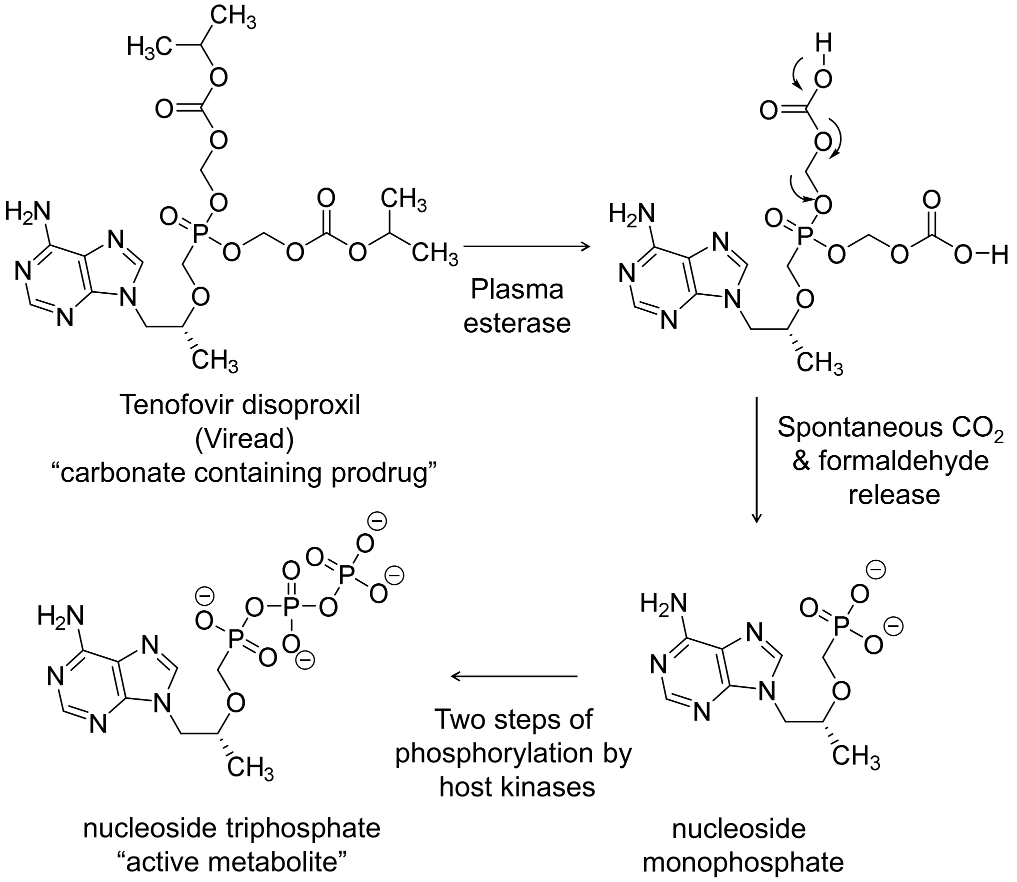

Figure S1. The chemical structure of tenofovir disoproxil and schematic representation of its metabolic bioactivation. Tenofovir is acyclic nucleotide derivative. It is also a prodrug. The corresponding triphosphate form is the active form and the inhibitor of the viral polymerase.

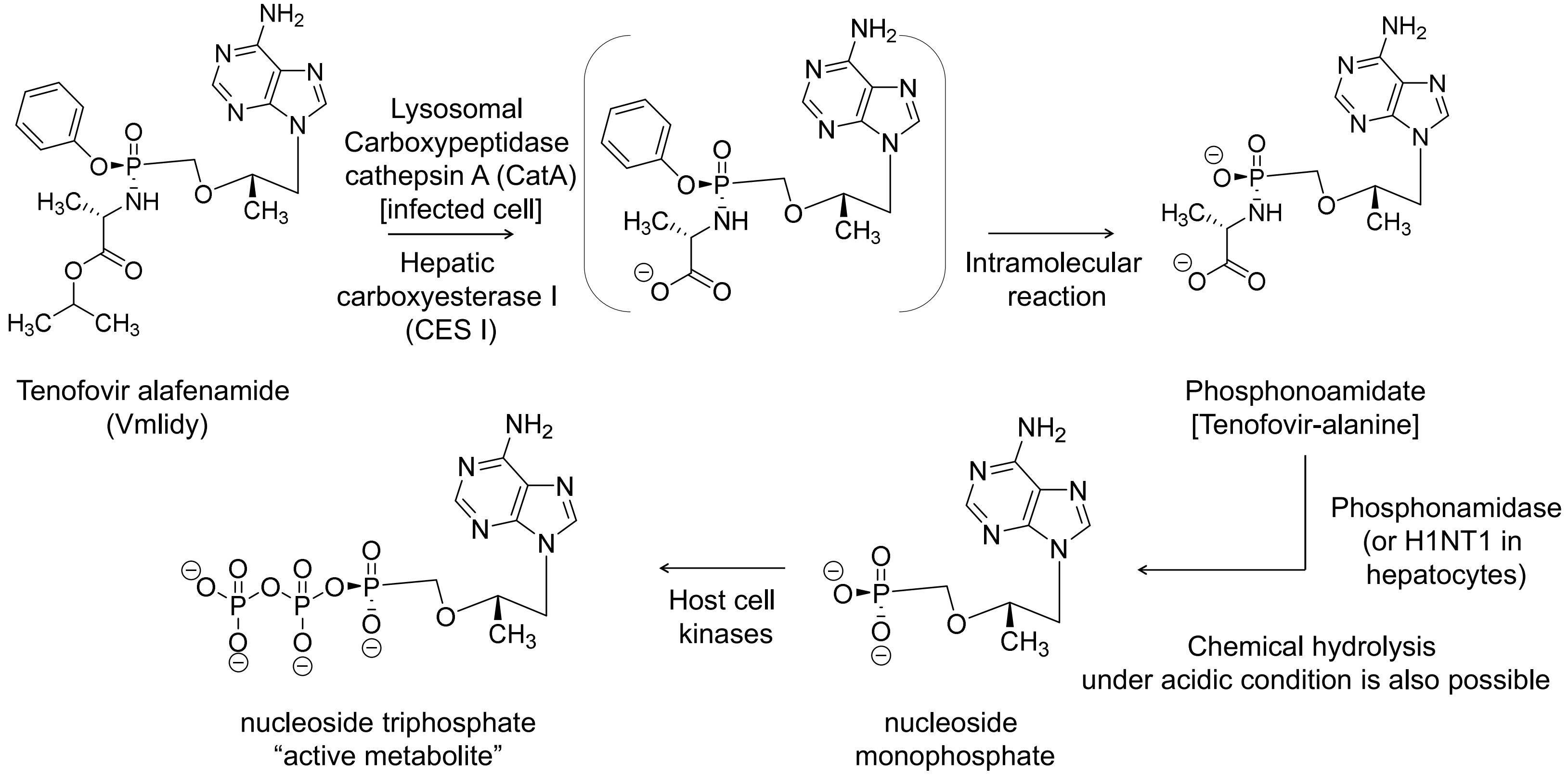

Figure S2. The chemical structure of tenofovir alafenamide and schematic representation of its metabolic bioactivation. Tenofovir is acyclic nucleotide derivative. It is also a prodrug that is more selectively activated in infected cells. The corresponding triphosphate form is the active form and the inhibitor of the viral polymerase.

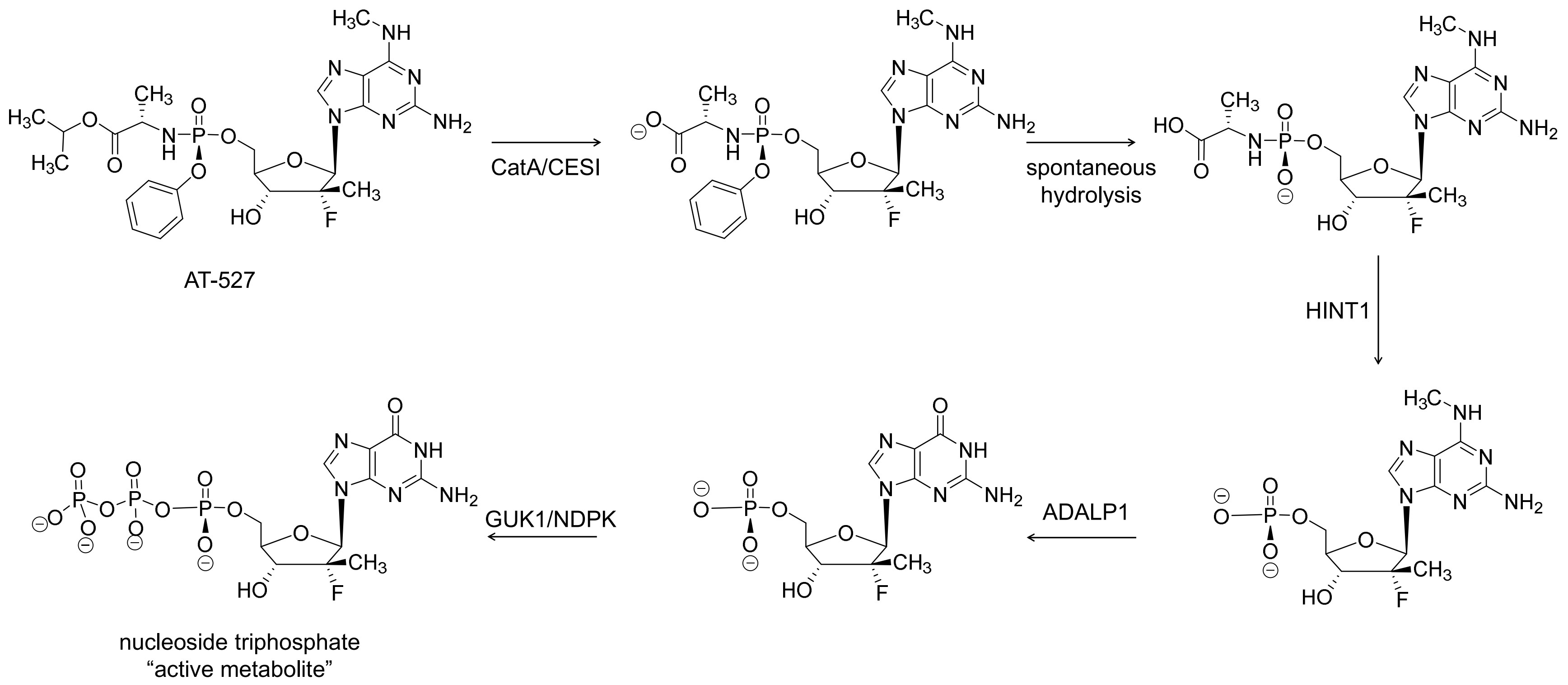

Figure S3. The chemical structure of AT-527 and schematic representation of its metabolic bioactivation. The corresponding triphosphate form is the active form and the inhibitor of the viral RNA polymerase.

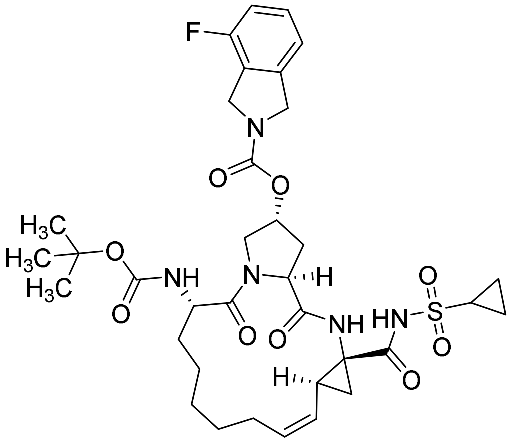

Danoprevir
(Ganovo)

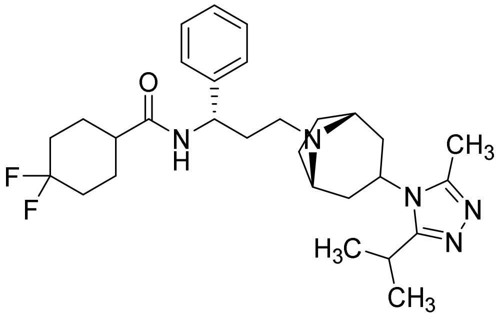

Maraviroc
(Selzentry)

Figure S4. The chemical structure of danoprevir and maraviroc. The former is macrocyclic peptidomimetic antiviral drug. It is inhibitor of NS3/4A HCV protease, an important processing enzyme complex. The latter is azabicyclic molecule that exhibits antiretroviral activity by blocking the interaction between HIV-1 gp120 and CCR5 on human CD4-presenting cells, that is necessary for HIV-1 to enter cells. It was also shown that maraviroc may potentially act as a potential inhibitor of M^{pro}.

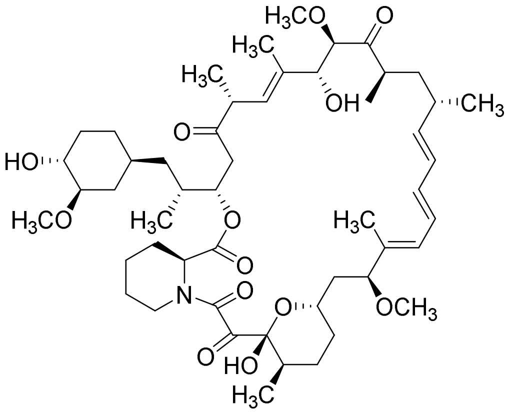

Sirolimus
(Rapamune)

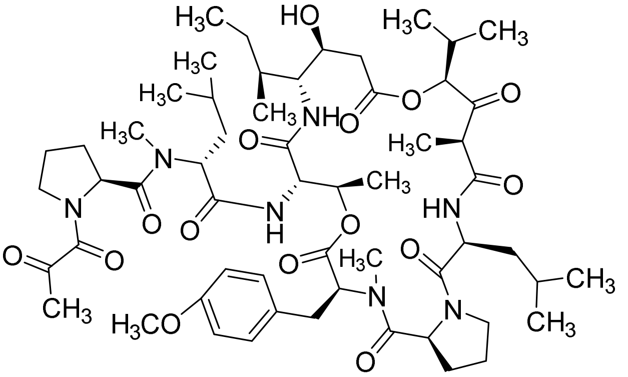

Plitidepsin
(Aplidin)

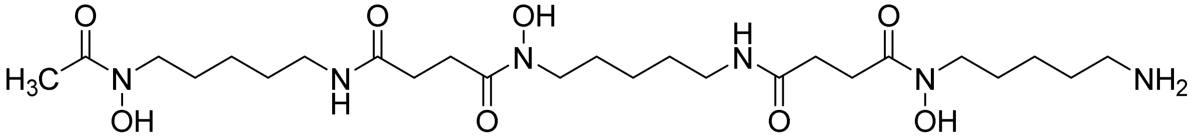

Deferoxamine
(Desferal)

Figure S5. The chemical structures of three natural products that are currently being tested against COVID-19. Sirolimus is an immunosuppressive agent and mTOR pathway inhibitor. Plitidepsin is targeting eukaryotic translation elongation factor 1 alpha 1. Deferoxamine is antiviral and anti-inflammatory agent.

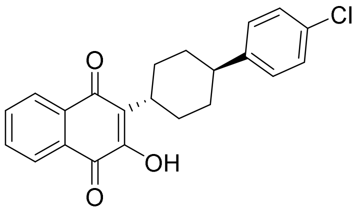

Atovaquone
(Mepron)

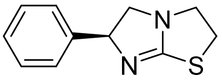

Levamisole

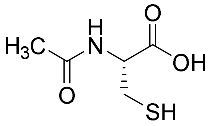

N-Acetylcysteine
(Acetadote)

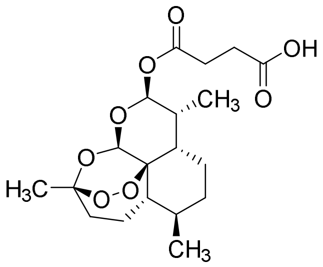

Artesunate

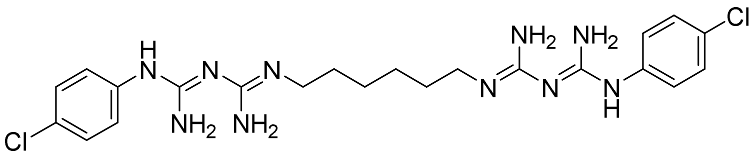

Chlorhexidine
(Peridex)

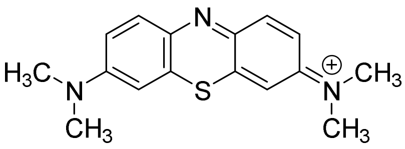

Methylene Blue
(ProvayBlue)

Figure S6. The chemical structures of various agents with potential antiviral activity attributed to various mechanisms. The list includes antimalarial drugs (atovaquone and artesunate), anti-inflammatory drug (levamisole), mucolytic agent (*N*-acetylcysteine), broad-spectrum antimicrobial agent (chlorhexidine), and a drug to treat methemoglobinemia (methylene blue).
